# Supplementary material for: Structural insights into the coupling mechanism of vectorial CO2 uptake by DAB1
Source: Proc Natl Acad Sci U S A. 2026 Oct 12;123(42):e2616712123. doi: 10.1073/pnas.2616712123 (PMC13684180; doi:10.1073/pnas.2616712123)
Supplement: Supplementary file 1 [file pnas.2616712123.sapp.pdf]

PNAS

Supporting Information for
Structural Insights into the Coupling Mechanism of Vectorial CO_2 Uptake by DAB1

Authors
Naiya R. Phillips[1], Luke M. Oltrogge[1,2], Jonathan P. Remis[3], David F. Savage[1,2,4*]

[1]Department of Molecular and Cell Biology, University of California; Berkeley, California 94720, USA;
[2]Howard Hughes Medical Institute, University of California; Berkeley, California 94720, USA;
[3]Department of Physics, University of California, Berkeley; Berkeley, CA 94720, USA;
[4]Innovative Genomics Institute, University of California; Berkeley, California 94720, USA;
*Corresponding author, Email: savage@berkeley.edu

This PDF file includes:

Figures S1 to S15
Tables S1 to S6
Legends for Movie S1

Other supporting materials for this manuscript include the following:

Movie S1

Figures

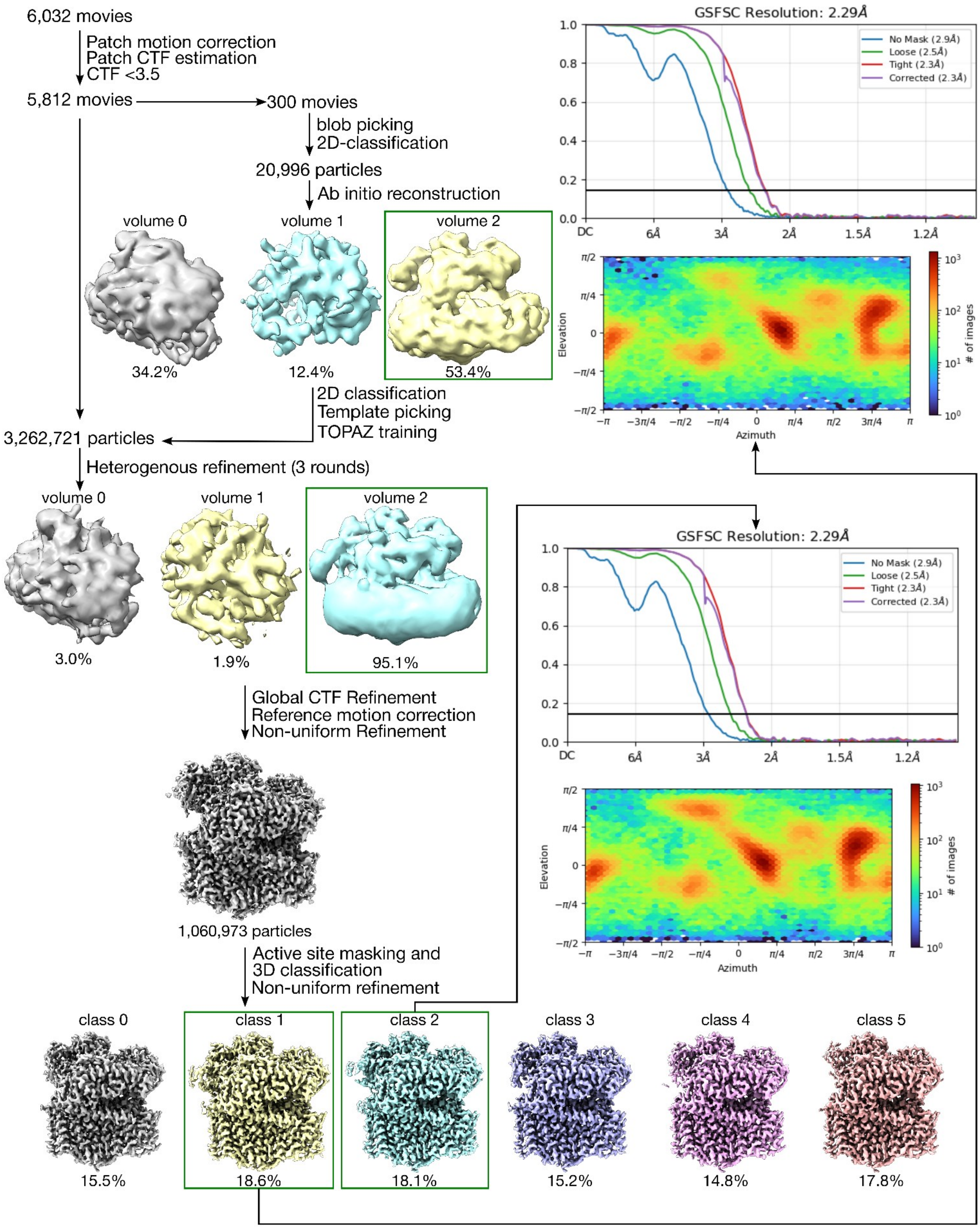

Fig. S1. Cryo-EM Image Processing workflow. Classes 1 and 2 were chosen for containing the best density surrounding the active site. Class 1 generated the State B model and Class 2 generated State A.

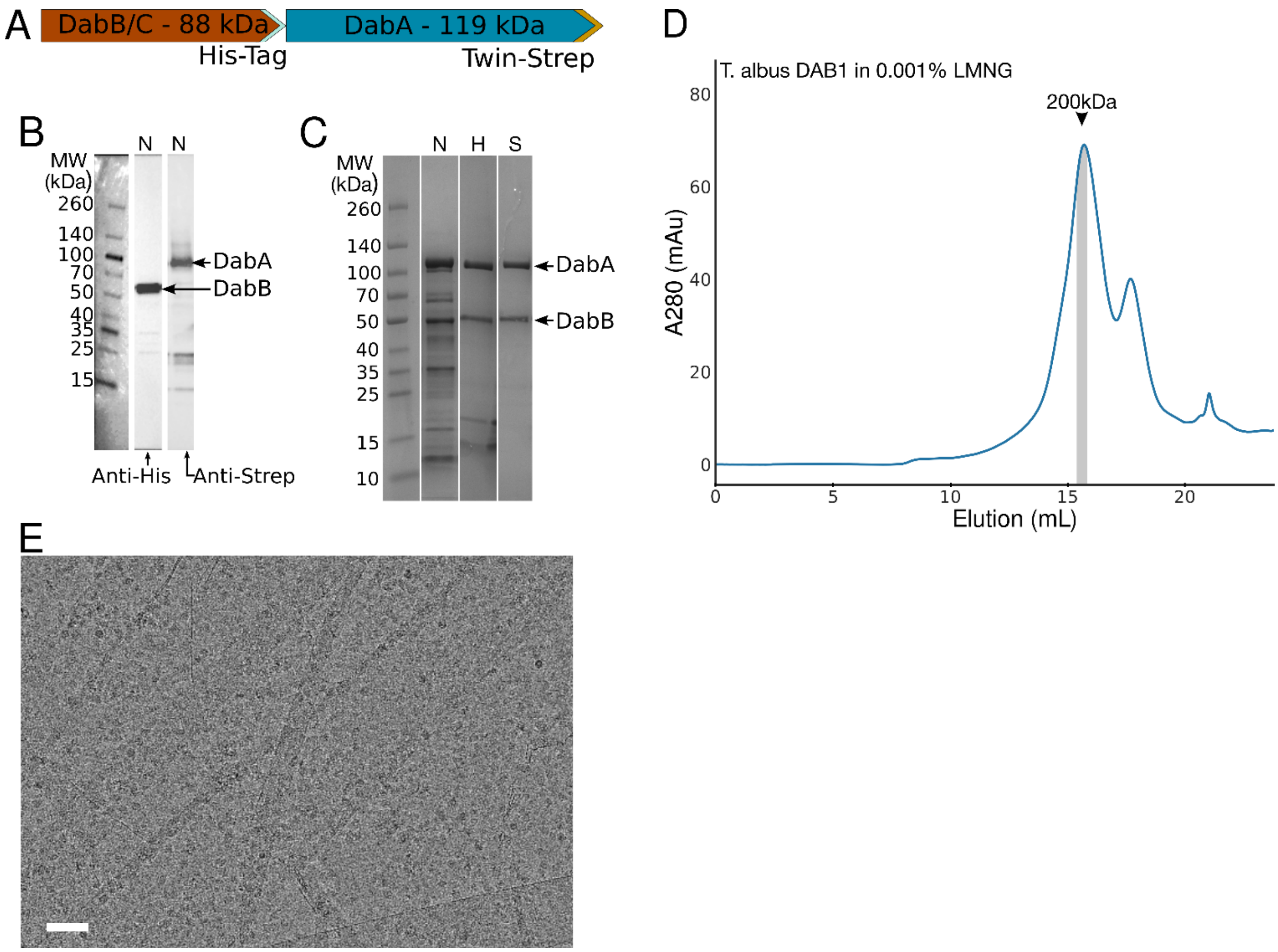

Fig. S2. Ni-IMAC and size exclusion chromatography purification of DAB1 complex. (A) Construct design for western blot analysis and purification of the DAB1 complex. Note that while a Twin-Strep tag was used to confirm the presence of DabA1, it was not used for affinity chromatography. (B) Western blot analysis of Ni-IMAC elution fraction confirming the presence of both DabA and DabBC after Ni-IMAC purification. N designates the fraction as a Ni elution fraction. (C) SDS-PAGE of purified enzymes after Ni-IMAC (N), 1 hour 70°C heat treatment (H) and size exclusion chromatography (S). (D) Size exclusion chromatogram of purified enzyme on a Superose 6 10/300 GL column equilibrated with 0.001% LMNG. (E) Representative micrograph of a graphene oxide grid containing purified DAB1 complex. Scale bar shows 50 nm.

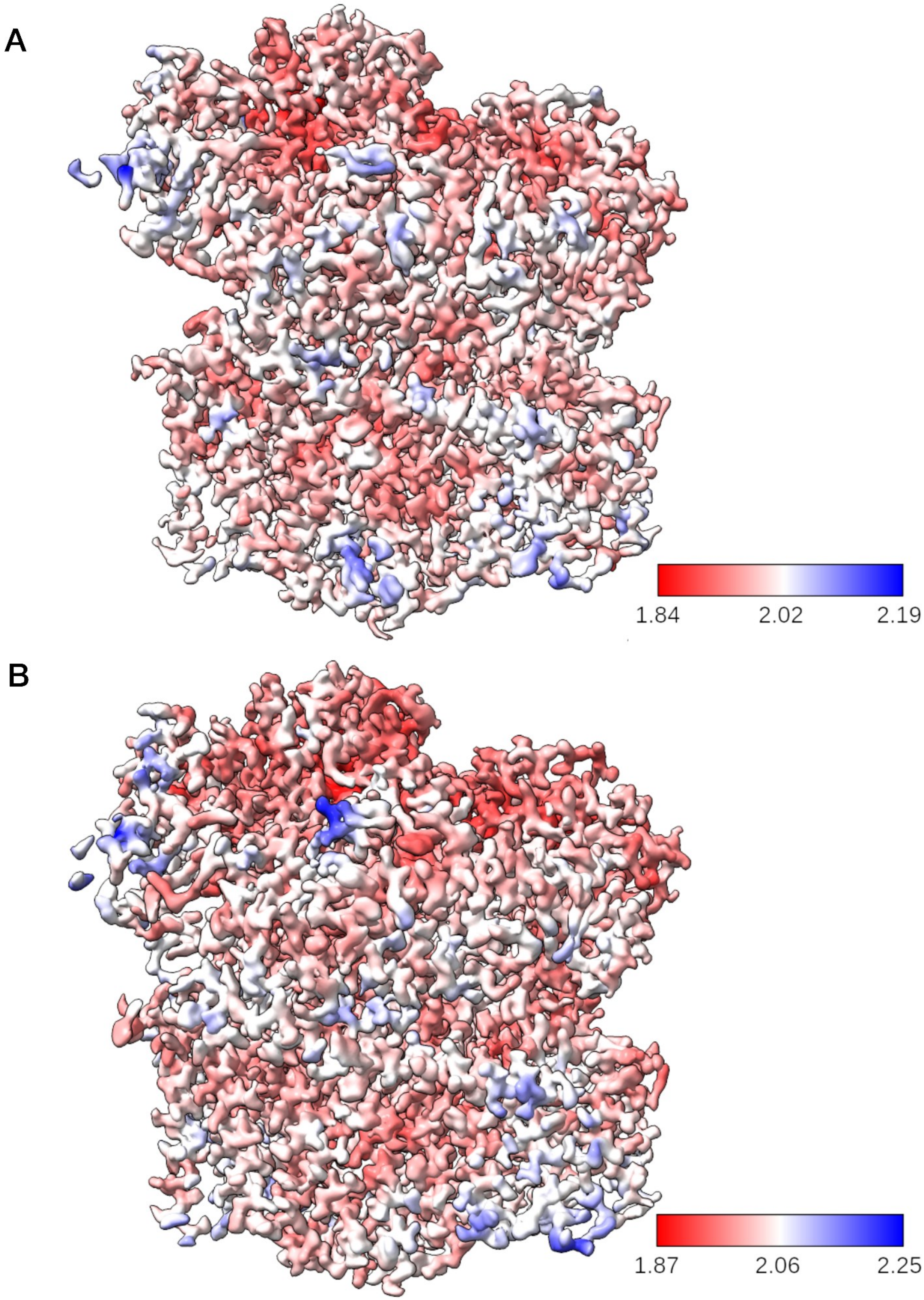

Fig. S3. Local resolution of the two conformations of the DAB complex. (A) State A (B) State B. Coloring generated in ChimeraX from the resolution estimates supplied by phenix.map_sharpening. Maps

are shown at a threshold of 0.5.

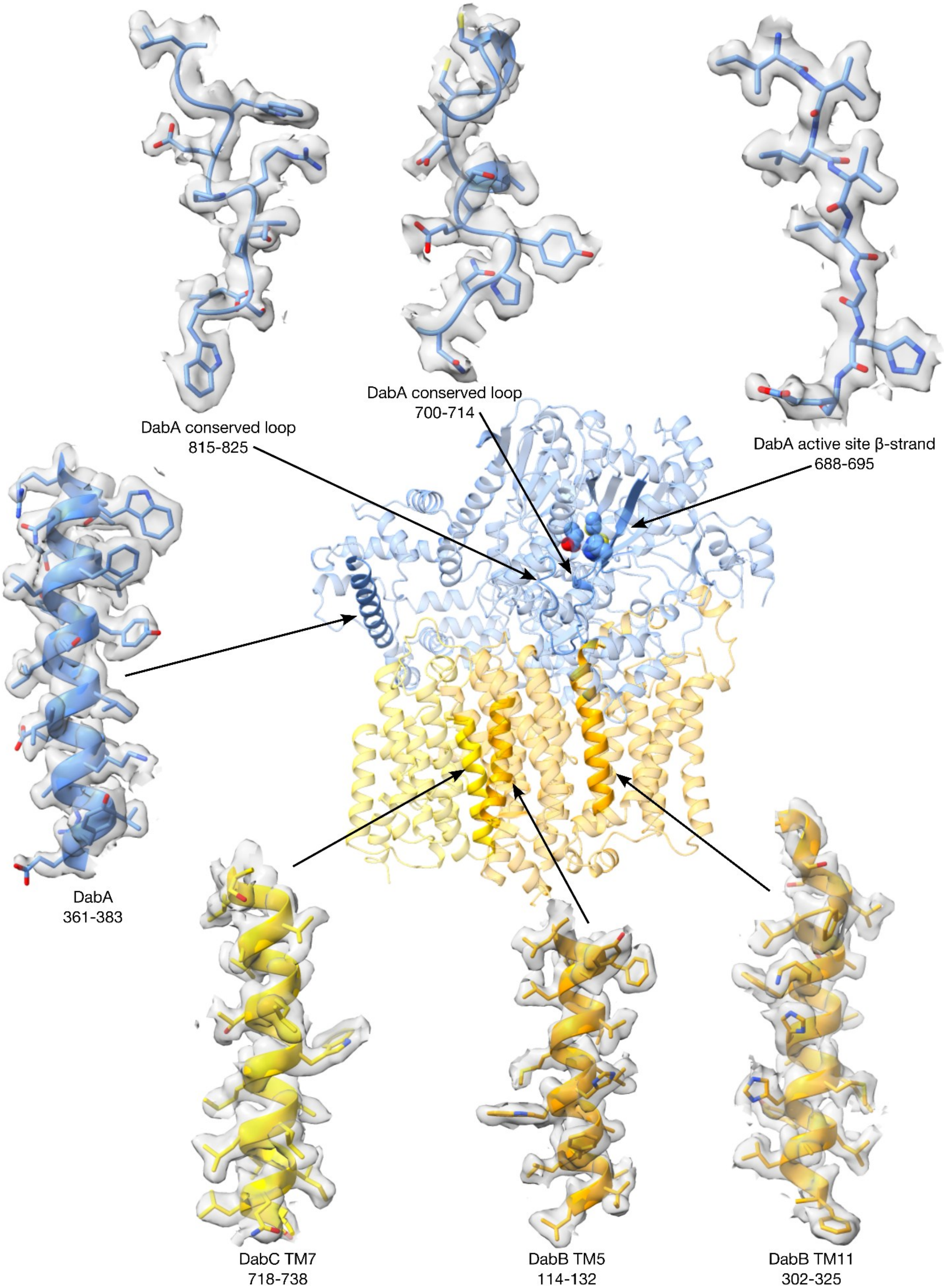

Fig. S4. Representative density fitting in DabB and DabA. Map and model of the State B conformation shown. Map threshold is set to 0.37.

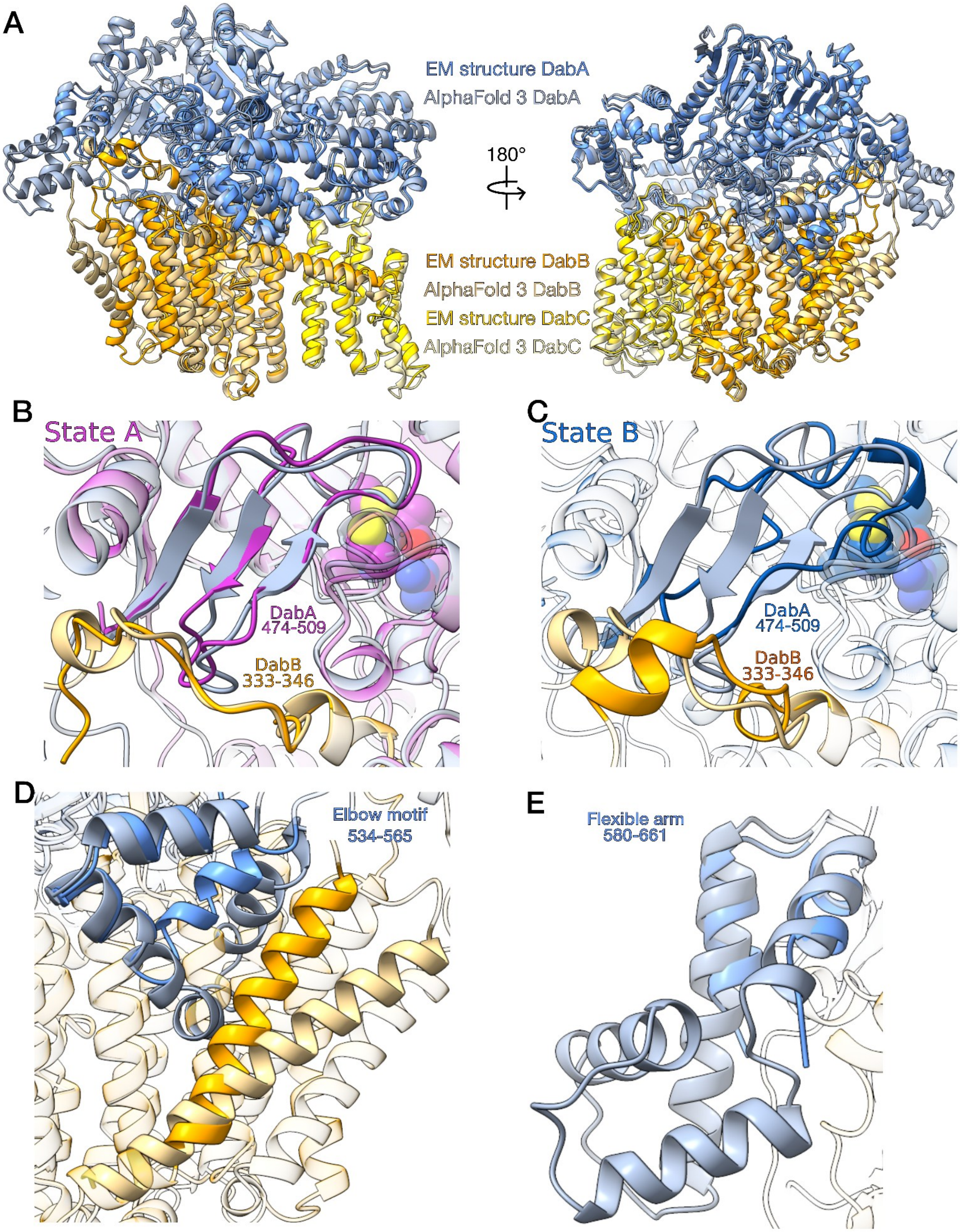

Fig. S5. Comparison of the experimentally determined DAB1 maps with the AlphaFold3 generated model. (A) Overlay of the two structures shown with AlphaFold3 model colored in a lighter color than the

cryo-EM model. (RMSD between 524 pruned atom pairs is 0.67 Å; across all 588 pairs: 5.20 Å) (B) View of lid domain in the State A conformation and (C) State B conformation. (D) View of the elbow motif. (E) AlphaFold 3 structural prediction of the flexible arm domain that we were unable to visualize at high resolution in our maps.

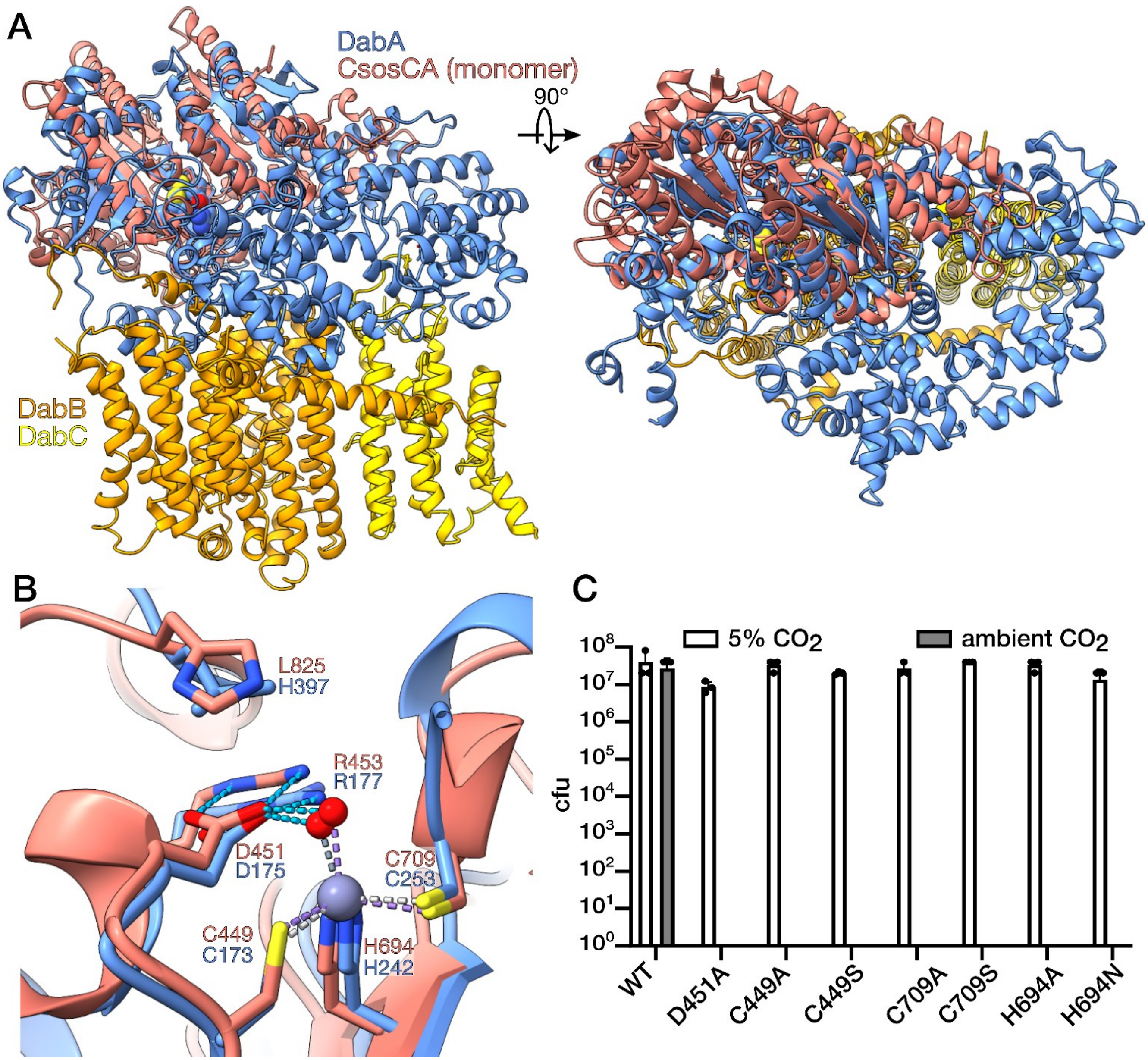

Fig. S6. Comparison of experimental *T. albus* model with CsosCA. (A) Structural overlay of CsosCA (PDB:2FGY) and DAB1 (State A conformation) (RMSD between 45 pruned atom pairs is 1.27 Å; across all 258 pairs: 12.55 Å) (B) View of the conserved active site structure, with the notable exception of L825. (C) Growth of CAFree expressing active site mutants in 5% CO_2 (permissive) and ambient air (selective). Any mutation of these residues abolishes DAB activity, and no colonies are observed in ambient air.

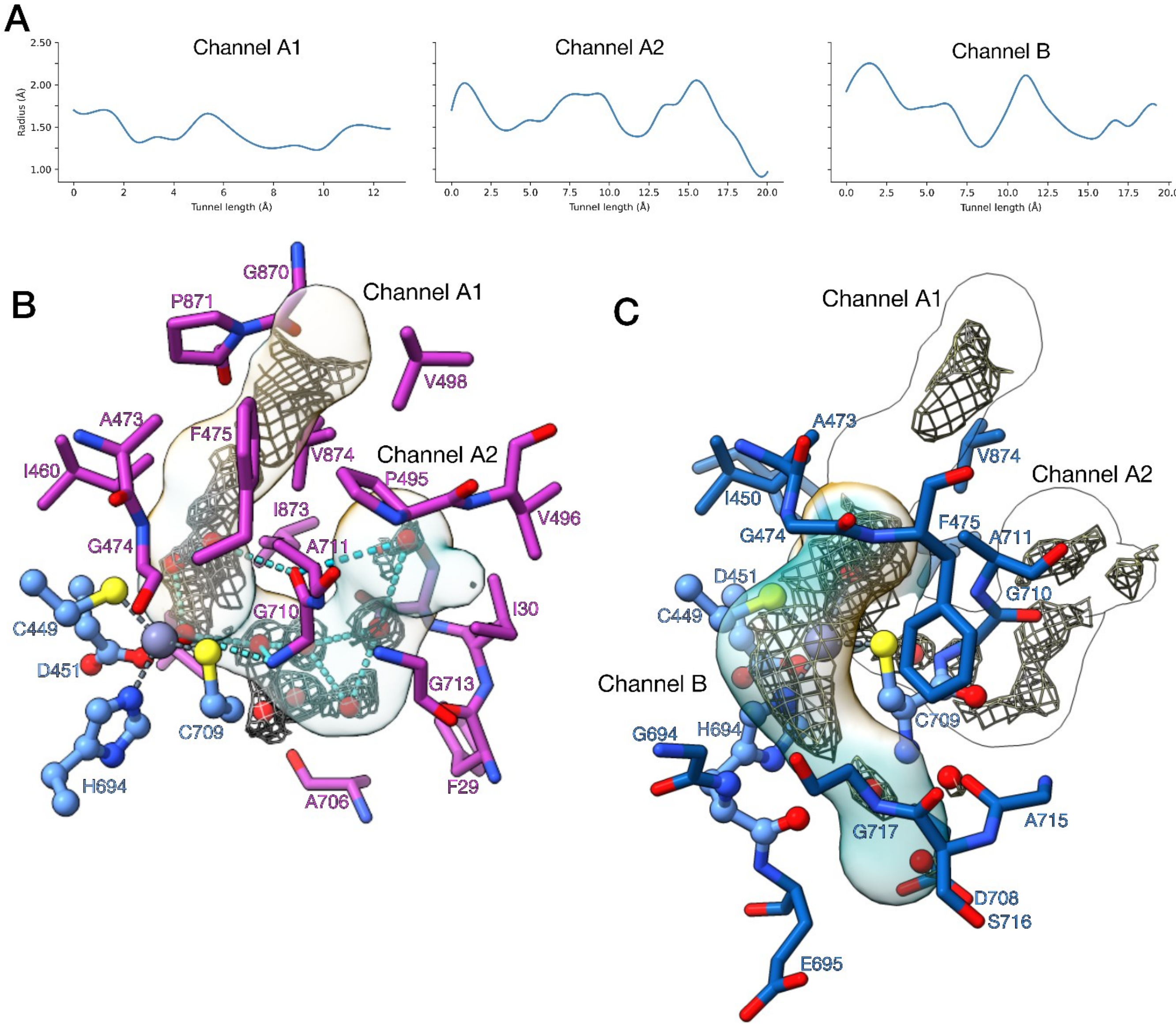

Fig. S7. CAVER analysis of State A and State B conformations with internal electron density. (A) Bottleneck profiles of each channel. (B) State A conformation. Both the CO_2 (Channel A1) and H_2O (Channel A2) channels are shown with internal electron density. Channel A2 contains distinct density blobs assigned as waters while Channel A1 shows a dispersed density. C) State B conformation. Only the hydrophilic Channel B is open, though it also contains dispersed density. Density likely from water molecules still resides in Channel A2 in this state.

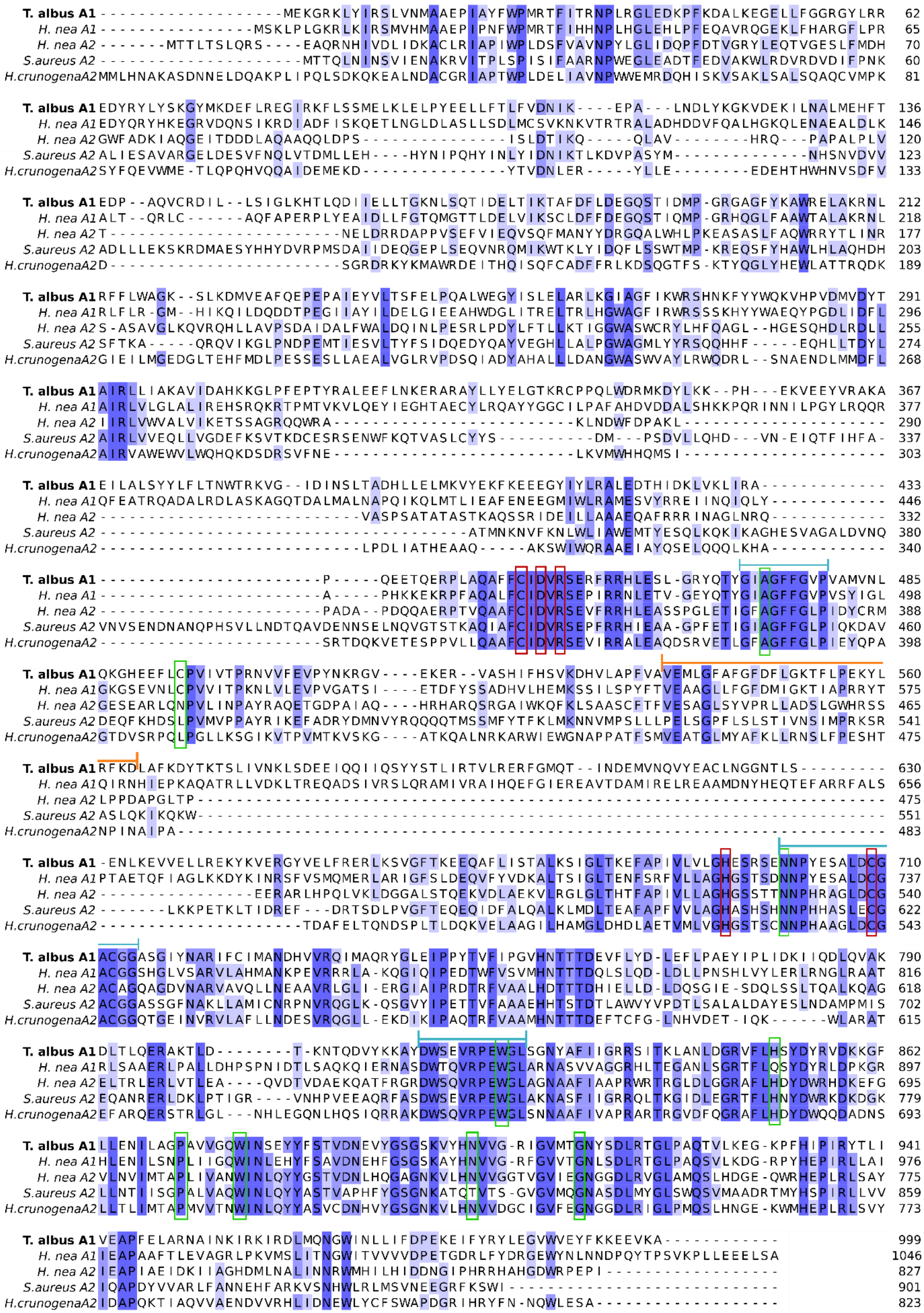

Fig. S8. Sequence conservation of *T. albus* DabA1 compared to other DabA homologs that have been well-characterized. Active site residues boxed in red and residues highlighted in the error-prone

PCR screen boxed in green. Elbow motif indicated by an orange bracket. Highly conserved motifs in DabA1 homologs indicated by blue brackets.

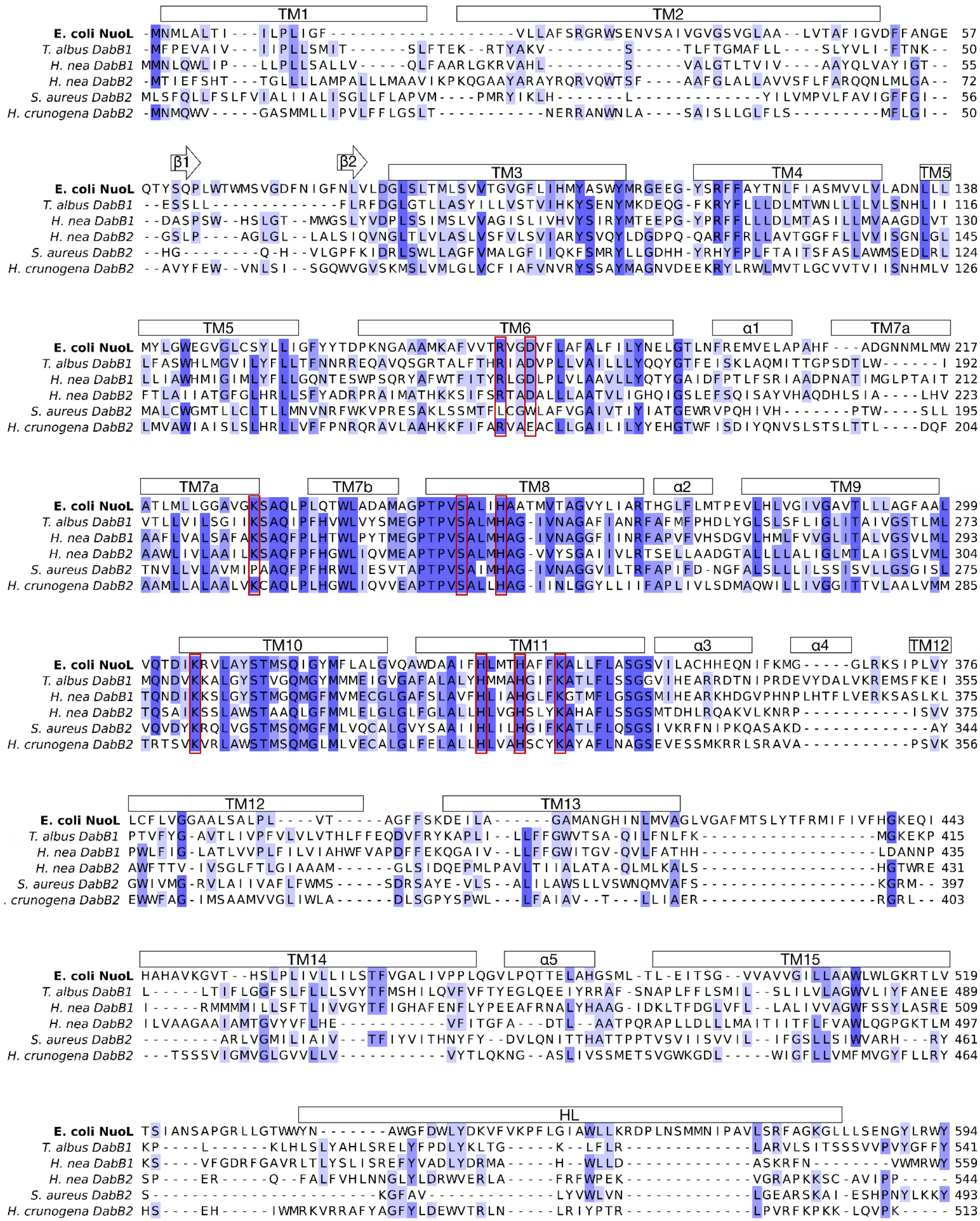

Fig. S9. Sequence conservation of *T. albus* DabB1 compared to *E. coli* NuoL and other DabB homologs that have been well-characterized. Proton translocation residues boxed in red. Secondary structure of the *T. albus* DabB1 are shown above. HL is the lateral helix.

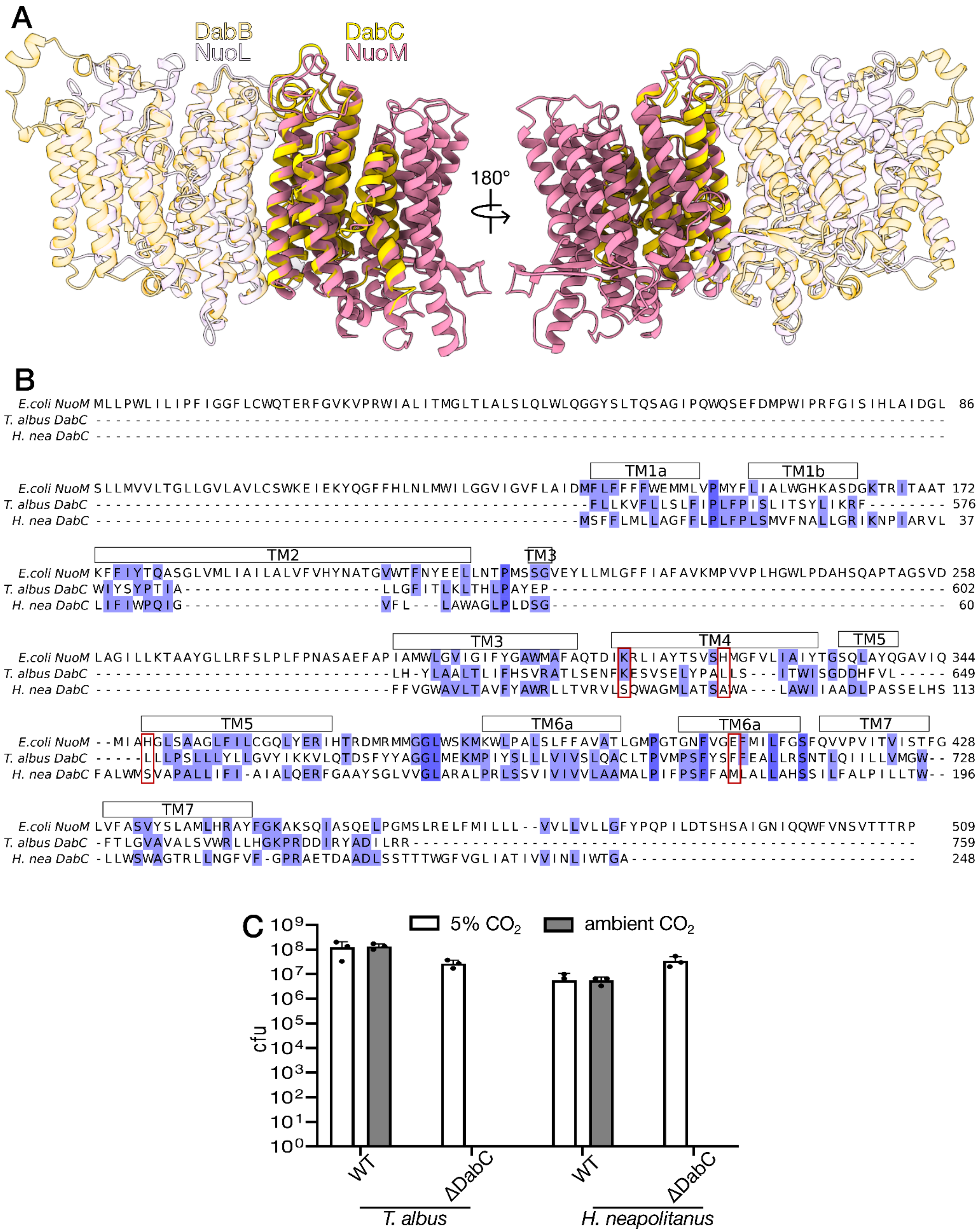

Fig. S10. Structural and sequence homology of DabC. (A) Structural overlay of *T. albus* DabBC and *E. coli* Complex I subunits NuoL and NuoM (PDB: 7Z7s). DabB and NuoL are shown as partially

transparent while DabC and NuoM are shown in full color. (B) The MSA of *E. coli* NuoM and two DabA1 homologs shows very little sequence homology despite the structural alignment. Proton translocation residues in NuoM (boxed in red) are substituted mostly for nonpolar residues in DabA1. (C) Growth of CAFree expressing *ΔdabC* constructs of DAB1 homologs in 5% CO_2 (permissive) and ambient air (selective). No growth is observed in air when DabC is deleted.

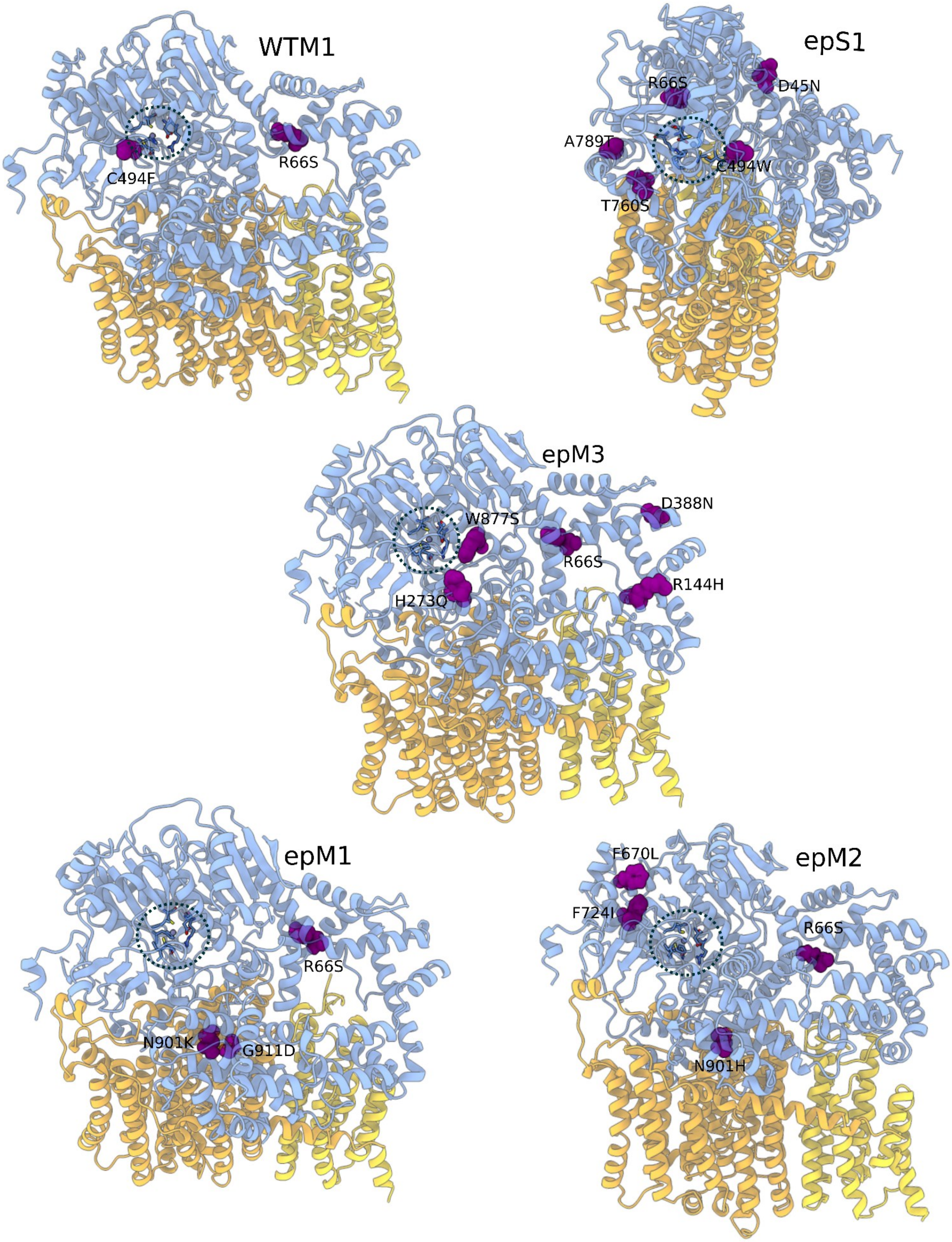

Fig. S11. Models showing the residues mutated in variants isolated from the epPCR screen of *T. albus* Dab1. Mutated residues shown as purple spheres in the context of the full structure. The active site is circled by the dashed line.

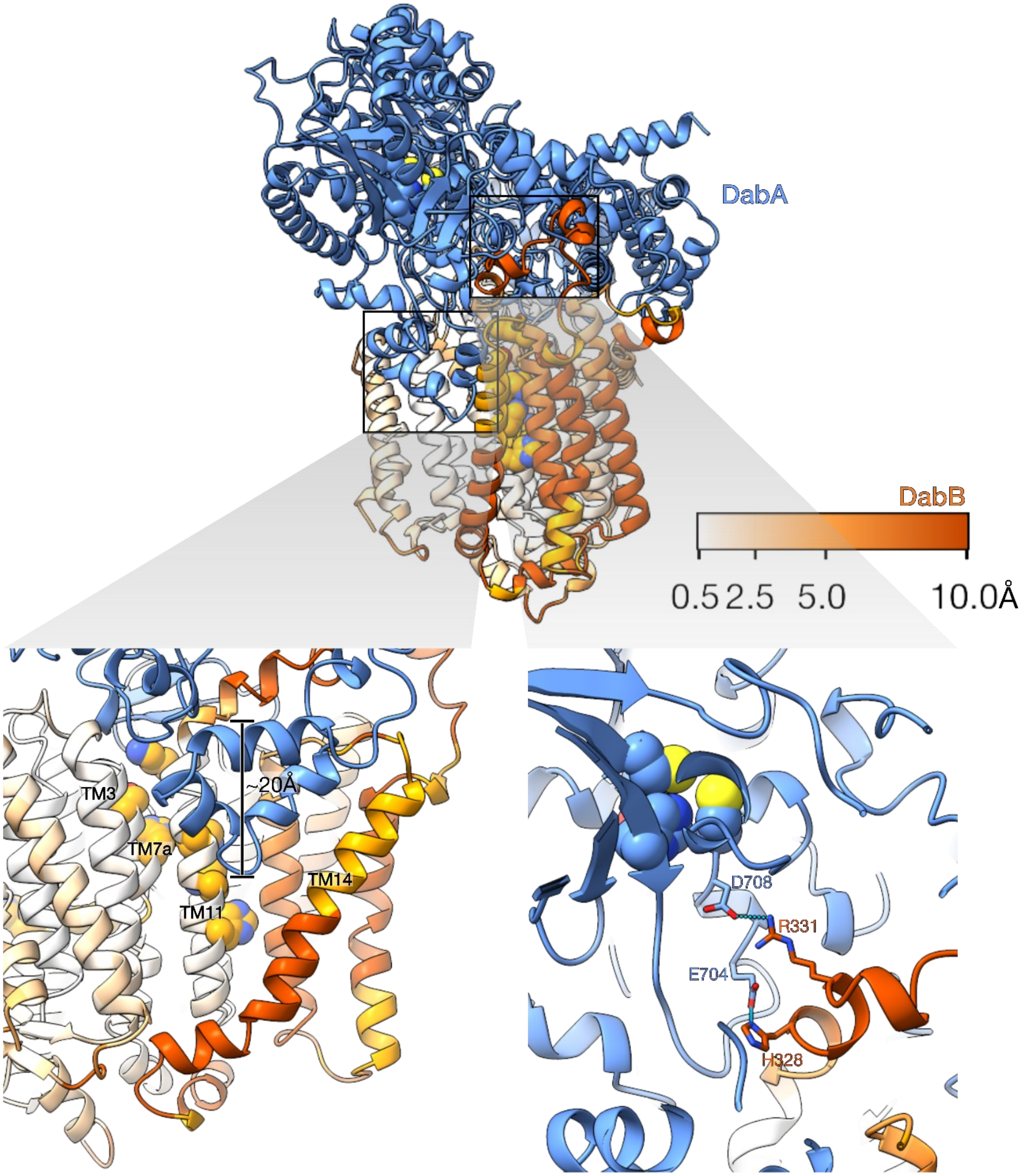

Fig. S12. DabA and B swap helices to transmit conformational changes. Model is State B in which DabB is colored according to RMSD value to *E. coli* NuoL. Insets show the disruption in the DabB TM helices caused by the elbow motif, and the helix DabB extends to interact with the DabA active site.

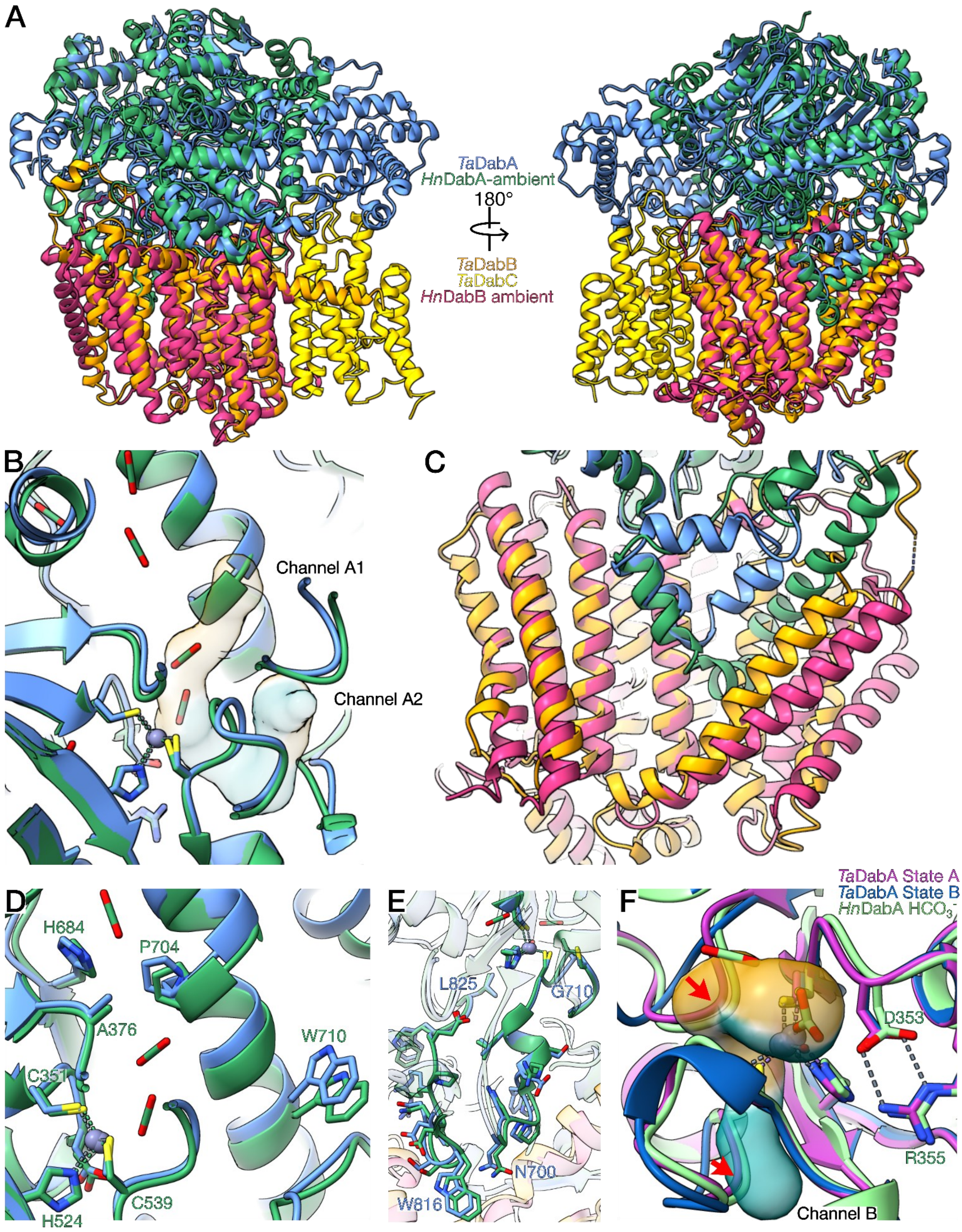

Fig. S13. Comparison of *T. albus* DAB1 and *H. neapolitanus* DAB2 complexes. (A) Overlay of State A of TaDAB1 complex and the HnDAB2 complex in ambient air (PDB:9RD0). The DAB1 complex is larger, containing DabC (shown in yellow) as well as additional domains in DabA which binds the

cytosolic face of DabC. (B) The active site structure is similar between the two complexes, and the C1 and C2 CO_2 molecules observed in the HnDAB2 structure are found within the proposed CO_2 channel A1. (C) The elbow motif differs in shape between the two complexes: in DAB1 it does not extend as far into the membrane or the interior of DabB. (D) Key decoupling residues found in the suppressor screen align well between TaDAB1 and HnDAB2. (E) The structure of the conserved motifs between DabA and DabB is well conserved between TaDAB1 and HnDAB2. (F) Overlay of the structures of TaDabA in State A (magenta) and State B (blue) and HnDabA bound to HCO_3^-(PDB:9RD8, green). Even when bound to HCO_3^-, DAB2 more resembles State A in which the proposed HCO_3^- release channel (Channel B) is closed, as indicated by red arrows.

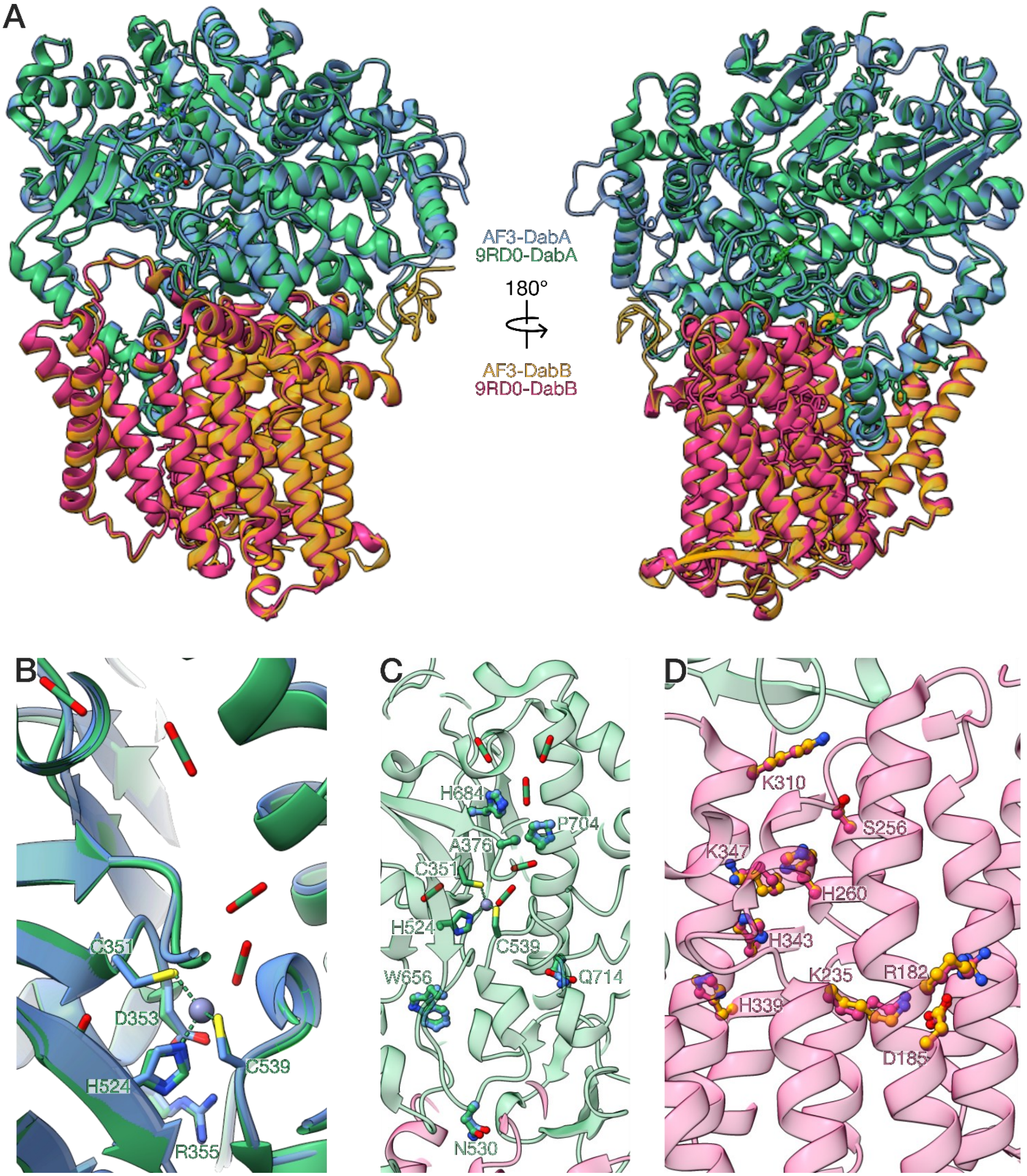

Fig. S14. Comparison of the experimental *H. neapolitanus* DAB2 structure with the predicted AlphaFold 3 structure shows overall agreement. (A) Overlay of the experimental DAB2 model (PDB:9RD0) and the predicted AF3 model. (B) Comparison of the active site shows excellent agreement between the predicted and experimental model. (B) The orientation of residues found to be important to coupling in our error-prone PCR screen are well-predicted by AF3. (C) The positions of conserved proton translocation residues in DabB were accurately predicted by AF3.

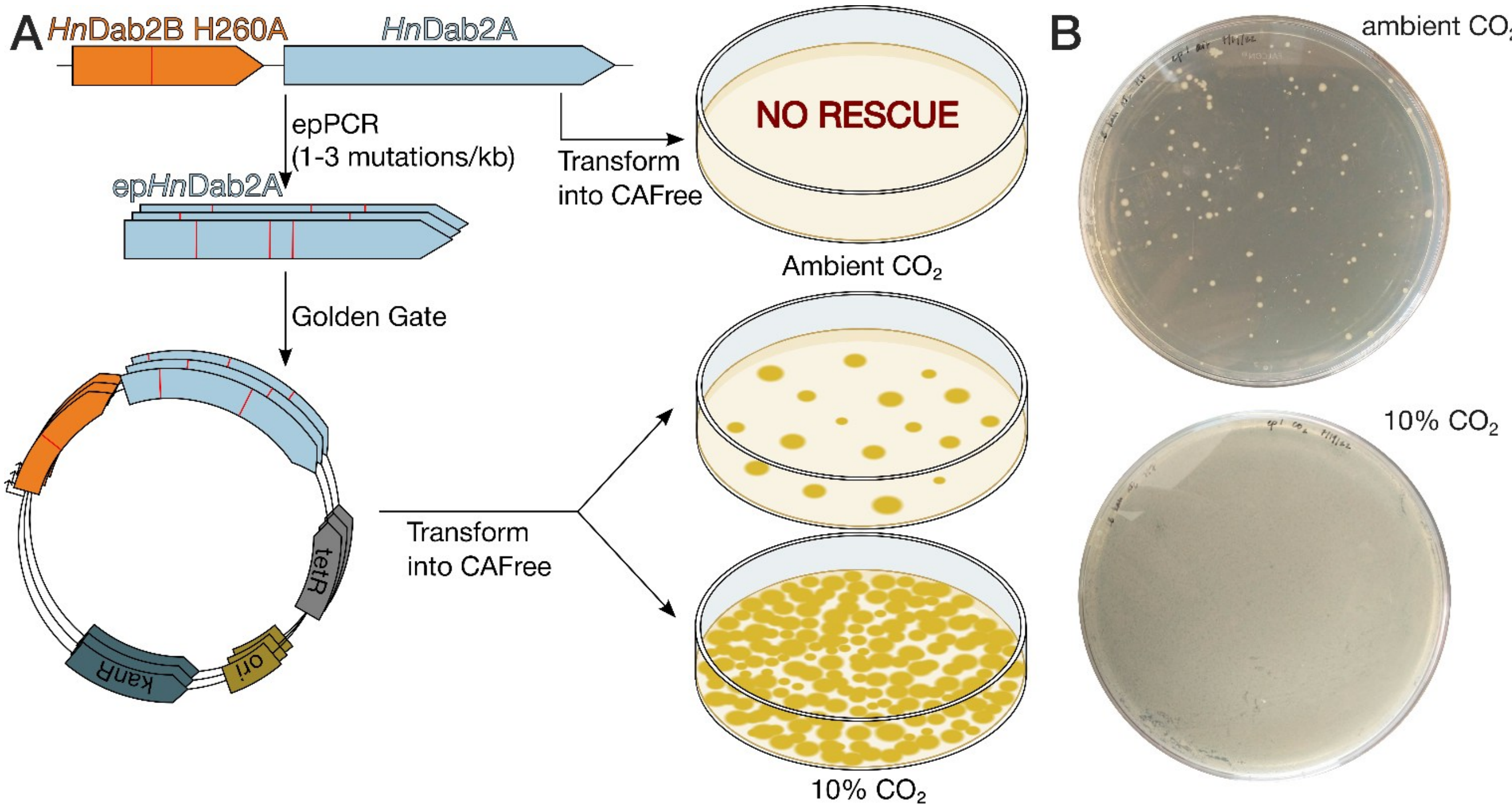

Fig. S15. Suppressor screen performed on *H. neapolitanus* DabA. (A) Schematic of the suppressor screen used to identify mutants in which DabA CA activity is decoupled from DabB proton translocation in the HnDAB2 complex. (B) Representative plates showing individual colonies under selective conditions (ambient air) and a lawn under permissive conditions (10% CO2).

Tables

Table S1. Cryo-EM data collection, refinement, and model building statistics.

	**State A**	**State B**
**Data Collection and Processing**		
Microscope	Krios G3	Krios G3
Detector	GATAN K3	GATAN K3
Nominal magnification	81,000	81,000
Accelerating Voltage (kV)	300	300
Electron exposure (e-/Å²)	50	50
Nominal defocus range (μm)	-1.5 to -1.8	-1.5 to -1.8
Pixel size (Å)	0.525	0.525
Symmetry imposed	C1	C1
Initial particle images (no.)	3,262,721	3,262,721
Final particle images (no.)	164,827	168,739
Map resolution (Å)	2.12	2.13
FSC threshold	0.143	0.143
Map resolution range (Å)	1.84-2.19	1.87-2.25
Map sharpening B factor (Å²)	phenix.map_sharpening	phenix.map_sharpening
**Model Composition**		
Non-hydrogen atoms	13789	14199
Protein Residues	1647	1678
Water	649	618
Zn^{2+}	1	1
**Model Validation**		
Bonds (RMSD)		
Length (Å)	0.013	0.012
Angles (°)	1.51	1.33
MolProbity score	2.22	2.12
Clash score	8.15	7.74
Ramachandran plot (%)		
Outliers	0.31	0.6
Allowed	4.65	3.54
Favored	95.05	95.86
B-factors (mean)		
Protein	28.24	31.56
Ligand	44.89	62.98
Water	28.04	29.21
**Model to map fit**		
CC (mask)	0.84	0.84
CC (box)	0.66	0.65
CC (peaks)	0.69	0.68
CC (volume)	0.81	0.81

Table S2. *H. neapolitanus* variants isolated with uncoupled CA activity.

Variant	Non-synonymous Mutations present
WT1	Q714K
WT2	A376S
WT3	Q714E
epB1	N530D
epB2	Q714K
epB3	P704S
epS1	P398S, L623M
epS2	V652L, A659G, Q714E
epS3	R258H, W656C
epB4	I175F, P337A, G540C
epS4	A29T, H249R, I256V, S270R, N530S
epS5	Q714R
epS6	H110L, W456C
epM1	S178M, Q714E
epM2	R622L, W656R
epM3	W656R
epM4	H684R
epM5	G540C

Table S3. Primers used in this study

Primer number	Primer purpose	Primer name	Primer sequence
126	pFE backbone amplification	jjd019bbGG-F	gactgaGGTCTCtgaagcggaattcctgc
127		jjd019bbGG-R	gactgaGGTCTCggacggtacctttctcctct
128	gblock amplification	T albus DabB F	GATCAAGGTCTCCCGTCCATG
129		T albus DabB R	AGCTTCggtctcCATAGGGTTACCTCCGTAGAATGTCTG
130		T albus DabA F	GATCAAGGTCTCCCTATGGAGAAAGGA
131		T albus DabA R	GATCACggtctcGCTTCATCACGTACCGCCG
128		A acido DabB F	GATCAAGGTCTCCCGTCCATG
132		A acido DabB R	TCATTGggtctcCGTCACTTCAAAATCGCCTCCTTCA
133		A acido DabA F	CAGCTAGGTCTCGTGACGC
134		A acido DabA R	GCGCATggtctcGCTTCAACCTCTCGCGCTTG
128		F thermo DabB F	GATCAAGGTCTCCCGTCCATG
135		F thermo DabB R	AGCTTCggtctcCTGCATAGAATGCTCCGACGC
136		F thermo DabA F	ATCCAGGGTCTCATGCAGACC
137		F thermo DabA R	GATCACggtctcGCTTCATTCTAATTCCAGGACTTGCCATTG
128		T marianensis DabB F	GATCAAGGTCTCCCGTCCATG
138		T marianensis DabB R	AGCTTCggtctcCTTCATCGCAAAATCGCCTCCTTC
139		T marianensis DabA F	CAGCTAGGTCTCATGAAGACCG
140		T marianensis DabA R	GATCACggtctcGCTTCAGCCCGCACG
141		A caldus DabB F	GATCAAGGTCTCCCGTCCGT
142		A caldus DabB R	AGCTTCggtctcGCTCATTGTACACCTCCAGATTGACC
143		A caldus DabA F	ACTGACGGTCTCATGAGCTCTAACAA
144		A caldus DabA R	GATCACggtctcGCTTCAAATTGATACCCACCGTCCTT
186	cloning T. albus Dab excluding pII	Ta-nopII-pFE-gg-R	ggctacGGTCTCgcttcaTCATGCTTTCACCTCC
187	DabB proton translocation mutants	H122A_F	cgtcatGGTCTCCGCGAGCTGGgcgCTGATGGGAG
188		H122A_R	cgtcatGGTCTCCTCGCGAACAGAATAATCAGATGGTTG
189		D156A_F	cgtcatGGTCTCCCGCATCGCAgcgGTACCGTTAC
190		D156A_R	cgtcatGGTCTCATGCGGTGGGTGAAAAGCG
191		K204A_F	cgtcatGGTCTCGGGAATCATTgcgTCAGCCCAAATTC
192		K204A_R	CGTCATggtctcATTCCCGAAAGAATAACCAGCAGAG

193		H211A_F	CGTCATggtctcAATTCCGTTCgcgGTATGGTTA GTGTATTCC
194		H211A_R	cgttgaGGTCTCGGAATTTGGGCTGACTTAAT GATTC
195		H229A_F	CGTCATggtctcCGCATTAATGgcgGCTGGTAT TGTTAATGCCG
196		H229A_R	CGTCATggtctcAATGCGCTTACCGGCGTC
197		H308A_F	CGTCATggtctcGGCGCTGTATgcgATGATGGC ACATGGTATC
198		H308A_R	CGTCATggtctcAGCGCCAGAGCGAATGCGCC A
199		H312A_F	CGTCATggtctcTATGATGGCAgcgGGTATCTTT AAAGCGACC
200		H312A_R	CGTCATggtctcATCATATGATACAGCGCCAG AG
201		K316A_F	CGTCATggtctcTGGTATCTTTgcgGCGACCCT GTTTC
202		K316A_R	CGTCATggtctcATACCATGTGCCATCATATGA TAC
203		Q381A_F	CGTCATggtctcGTTCTTCGAAgcgGACGTGTT CCG
204		Q381A_R	CGTCATggtctcAAGAACAGATGGGTTACCAG CAC
205		D382A_F	CGTCATggtctcCTTCGAACAGgcgGTGTTCCG CTATAAG
206		D382A_R	CGTCATggtctcTCGAAGAACAGATGGGTTAC CAG
283	error prone DabA hit single mutants	R66S-F	GATACAggtctcACGCGAGGATTATtcTTATCTG TATTCAAAGGG
284		R66S-R	GATACAggtctcTCGCGTCGCAGGTAGC
207		C494Y-F	cagtgaGGTCTCAGGAATTCTTATACCCAGTT ATCGTG
208		C494F-F	cagtgaGGTCTCAGGAATTCTTATTCCCAGTT ATCGTG
209		C494W-F	cagtgcGGTCTCAGGAATTCTTATGGCCAGTT ATCGTG
210		C494X-R	cagtcaGGTCTCATTCCTCATGACCTTTCTGC AAG
211		F670L-F	cagtcaGGTCTCGAGGAACAAGCTTTGCTGAT CAGTAC
212		F670L-R	cagtcaGGTCTCTTCCTCTTTGGTAAAGCCAA CC
213		F724I-F	cagtcaGGTCTCGCGCGCATTATCTGTATAAT GGC
214		F724I-R	cagtcaGGTCTCGCGCGCATTGTAAATACCG
215		T760S-F	cagtcaGGTCTCCAATACAACAAGCGACGAG GTG
216		T760S-R	cagtcaGGTCTCGTATTGTGCACACCGGGAAT AAATAC
217		A789T-F	cagtcaGGTCTCGATCTGCAGGTTACAAAAGA TTTAACAT
218		A789T-R	cagtcaGGTCTCCAGATCCTGTATGATCTTAT CTATCAAAGGG

219		R797C-F	cagtcaGGTCTCCATTACAAGAGTGTGCCAAGAC
220		R797C-R	cagtcaGGTCTCGTAATGTTAAATCTTTTGCAACCTGCAG
221		V819E-F	catgcaGGTCTCGTCCGAAGAGCGACCG
222		V819E-R	gactcaGGTCTCTCGGACCAGTCATAGGCTTTC
223		W877S-F	cagtcaGGTCTCGTGGGCCAATCGATTAACTCAGAG
224		W877S-R	cagtcaGGTCTCGCCCACCACGGCCGG
225		N901K-F	cagtcaGGTCTCGAAAGTTTATCACAAGGTTGTCGGG
226		N901H-F	cagtcaGGTCTCGAAAGTTTATCACCATGTTGTCGGG
227		N901X-R	cagtcaGGTCTCACTTTCGACCCAGAGCCATAAA
228		G911D-F	cagtcaGGTCTCGAGTCATGACTGACAACTATTCTGAT
229		G911D-R	cagtcaGGTCTCTGACTCCGATTCTCCCGAC
230	DabA Active Site mutants	C449A-F	TCAGTCggtctcACAAGCTTTCTTTgcCATTGACGTTCGG
231		C449S-F	TCAGTCggtctcACAAGCTTTCTTTTcaATTGACGTTCGG
232		C449X-R	TCAGTCggtctcGCTTGTGCTAACGGGC
233		D451A-F	TCAGTCggtctcTTTGCATTGcCGTTCGGTCAGA
234		D451N-F	TCAGTCggtctcTTTGCATTaACGTTCGGTCAGA
235		D451L-F	TCAGTCggtctcTTTGCATTctCGTTCGGTCAGAACG
236		D451X-R	TCAGTCggtctcTGCAAAAGAAAGCTTGTG
237		H694A-F	TCAGTCggtctcCCTTGTTCTGGGAgcTGAGTCCCGGTC
238		H694N-F	TCAGTCggtctcCCTTGTTCTGGGAaATGAGTCCCG
239		H694X-R	TCAGTCggtctcACAAGGACAATCGGAGCAAA
240		C709A-F	TCAGTCggtctcCGCCTTGGATgcCGGAGCATGC
241		C709S-F	TCAGTCggtctcCGCCTTGGATTcaGGAGCATGCG
242		C709X-R	TCAGTCggtctcAAGGCGCTTTCGTACG
243		C712A-F	TCAGTCggtctcTGCGGAGCAgcCGGTGGCGC
244		C712S-F	TCAGTCggtctcTGCGGAGCATcaGGTGGCGCGA
245		C712X-R	TCAGTCggtctcTCCGCAATCCAAGGC
285	DabC His tag	TaDabB-His-GG-F	gatacaGGTCTCaccatcaccatcaccatcacTAACCCTATGGAGAAAGGAAGAAAGCT
286		TaDabB-His-GG-R	gatacaGGTCTCgatggtgatggtgGCCACTAGAACCAGACCTCCGTAGAATGTCTGCAT

Table S4. Plasmids used in this study

Plasmid name	Description	Antibiotic
pJJD019	Inducible *H. neapolitanus* Dab2 operon in pFE backbone	kanamycin
pNRP034	inducible *T. albus* Dab1 operon including pII protein in pFE backbone	kanamycin
pNRP035	inducible *T. marianensis* Dab operon in pFE backbone	kanamycin
pNRP036	inducible *A. acidocaldarius* Dab operon in pFE backbone	kanamycin
pNRP037	inducible *F. thermophila* Dab operon in pFE backbone	kanamycin
pNRP038	inducible *A. caldus* Dab operon in pFE backbone	kanamycin
pNRP048	inducible *T. albus* Dab1 with a His tag on DabC and a Twin Strep tag on DabA in a pFE backbone	kanamycin
pNRP050	inducible *T. albus* Dab1 with no tags and no pII protein in pFE backbone	kanamycin

Table S5. TWIST gBlocks of codon-optimized DAB homologs.

Name	Insert Length	Insert Sequence
Thermo-crinis albus DabA	3383	GATCAAGGTCTCCCTATGGAGAAAGGAAGAAAGCTATACATAAGGT<br>CCCTTGTAAATATGGCCGCTGAGCCAATTGCCTATTTCTGGCCCAT<br>GAGAACCTTTATCACACGAAACCCTCTGCGCGGTTTAGAGGATAAG<br>CCTTTCAAAGACGCTCTTAAGGAAGGCGAACTCCTGTTCGGTGGG<br>CGTGGCTACCTGCGACGCGAGGATTATCGTTATCTGTATTCAAAGG<br>GTTATATGAAAGACGAGTTCTTGCGCGAAGGAATTCGTAAGTTCCT<br>CAGCTCGATGGAACTGAAGCTTGAACTGCCATATGAGGAGCTCTTG<br>TTCACATTATTCGTGGACAATATCAAGGAACCCGCGTTGAACGACC<br>TTTATAAGGGCAAGGTGGATGAAAAGATCCTTAATGCACTGATGGA<br>GCATTTCACGGAAGATCCAGCACAGGTATGTCGTGACATACTGCTG<br>TCTATTGGCCTCAAACACACCTTACAAGATATAATTGAGTTACTTAC<br>GGGCAAGAATTTAAGCCAGACAATTGACGAGTTAACAATTAAAACG<br>GCCTTCGACTTTCTTGATGAAGGGCAAAGCACCATTGACATGCCGG<br>GTCGTGGCGCGGGGTTCTATAAAGCATGGCGGGAACTCGCAAAGC<br>GGAACCTGCGATTCTTTCTCTGGGCGGGGAAATCTCTCAAGGACAT<br>GGTGGAAGCATTTCAGGAACCAGAGCCAGCCATCGAATACGTACT<br>CACCAGTTTCGAGTTGCCGCAGGCACTGTGGGAAGGCTATATTAG<br>CTTGGAGCTGGCGCGTTTGAAGGGAATCGCGGGATTCATCAAATG<br>GCGTTCGCATAATAAATTTTACTATTGGCAGAAGGTACATCCAGTC<br>GATATGGTTGACTATACGGCTATCCGCTTGCTGATCGCGAAAGCAG<br>TCATTGACGCTCATAAGAAAGGGCTGCCGTTCGAGCCAACATATCG<br>TGCCCTCGAAGAATTCCTTAATAAGGAAAGAGCTCGGGCATACCTG<br>CTGTATGAGTTGGGTACTAAGCGCTGTCCACCGCAATTATGGGATC<br>GGATGAAAGACTATTTGAAGAAACCCCATGAGAAGGTCGAAGAGTA<br>CGTTCGTGCCAAAGCGGAGATCCTTGCGCTTAGTTATTATTTATTCC<br>TGACGAATTGGACCCGGAAGGTTGGCATTGATATTAATTCTTTGAC<br>TGCCGACCATCTGTTAGAGCTGATGAAGGTCTATGAGAAATTTAAG<br>GAAGAAGAAGGCTATATTTATTTACGTGCGCTGGAAGACACGCATA<br>TCGACAAACTCGTGAAACTTATCCGTGCGCCACAGGAAGAAACGCA<br>GGAGCGCCCGTTAGCACAAGCTTTCTTTTGCATTGACGTTCGGTCA<br>GAACGCTTTCGCCGGCACCTGGAGTCTCTTGGGCGATACCAGACA<br>TATGGCATCGCAGGGTTCTTCGGAGTTCCGGTGGCAATGGTTAACT<br>TGCAGAAAGGTCATGAGGAATTCTTATGCCCAGTTATCGTGACCCC<br>GCGTAACGTAGTCTTCGAGGTCCCGTATAATAAGCGTGGCGTGGA<br>GAAAGAGCGGGTCGCTAGCCACATCTTTCACTCCGTCAAGGACCA<br>TGTTTTAGCTCCGTTCGTAGCTGTTGAGATGTTAGGTTTCGCGTTC<br>GGGTTCGACTTTCTGGGTAAAACGTTTCTCCCTGAAAAGTACTTAC<br>GGTTCAAAGACTTGGCATTCAAGGACTATACCAAGACCAGCTTGAT<br>TGTTAATAAACTGTCAGACGAGGAGATTCAACAAATCATTCAGAGTT<br>ATTACAGTACTCTGATTCGTACAGTTCTGCGGGAGCGTTTCGGCAT<br>GCAGACAATTAATGACGAAATGGTAAACCAAGTCTATGAGGCGTGT<br>CTGAACGGTGGTAATACCCTGAGTGAGAATTTAAAAGAGGTCGTGG<br>AGCTGCTGCGCGAAAAGTATAAGGTTGAACGGGGATACGTTGAGT<br>TGTTTCGGGAGCGCTTGAAGTCGGTTGGCTTTACCAAAGAGGAACA<br>AGCTTTTCTGATCAGTACCGCGCTGAAGTCAATCGGGCTTACGAAG<br>GAATTTGCTCCGATTGTCCTTGTTCTGGGACATGAGTCCCGGTCTG<br>AGAATAATCCGTACGAAAGCGCCTTGGATTGCGGAGCATGCGGTG<br>GCGCGAGCGGTATTTACAATGCGCGCATTTTCTGTATAATGGCAAA<br>CGACCATGTTGTTCGTCAAATTATGGCGCAGCGATATGGTCTTGAA<br>ATCCCGCCGTACACCGTATTTATTCCCGGTGTGCACAATACAACAA<br>CCGACGAGGTGTTTCTGTACGACTTAGAATTCCTGCCGGCTGAGTA<br>TATCCCTTTGATAGATAAGATCATACAGGATCTGCAGGTTGCAAAA<br>GATTTAACATTACAAGAGCGTGCCAAGACCCTGGACACGAAGAATA

		CCCAAGACGTTTATAAGAAAGCCTATGACTGGTCCGAAGTGCGACC<br>GGAGTGGGGCCTGAGTGGGAATTATGCATTTATTATTGGTCGTCGA<br>AGTATCACTAAGTTGGCTAACTTGGATGGCCGGGTATTTCTGCATT<br>CCTATGATTATAGAGTAGACAAGAAGGGATTCCTTTTGGAGAACATT<br>CTGGCTGGCCCGGCCGTGGTGGGCCAATGGATTAACTCAGAGTAT<br>TATTTTAGCACTGTTGATAATGAGGTTTATGGCTCTGGGTCGAAAGT<br>TTATCACAATGTTGTCGGGAGAATCGGAGTCATGACTGGCAACTAT<br>TCTGATTTACGCACCGGGCTGCCGGCTCAGACTGTTCTTAAGGAAG<br>GAAAGCCGTTTCATATCCCGATCCGTTATACTTTGATTGTTGAGGCA<br>CCTTTCGAACTGGCCCGCAACGCAATAAATAAGATCCGGAAAATCA<br>GAGACTTAATGCAGAATGGATGGATCAACCTTTTAATTTTCGACCCC<br>GAAAAGGAGATTTTCTACCGCTACTTAGAAGGTGTTTGGGTTGAAT<br>ACTTCAAAAAAGAGGAGGTGAAAGCATGAAAAGTGTAAAACTCTAT<br>GAGATGAAGAAGGTGGAAATAATCGTTAGAGGCGAAGATCTGGATT<br>TCGTGCTGGACCTGCTGGATCGCGCCGGAGCAACAGGTTATACAA<br>TTATCCACAATCTTAGTGGGAAGGGTTCCCATGGATTCCATGAGGG<br>GCATCTGTTGTTCAACGAGGAAGATACACTGGTGATGGTTATATCG<br>GTGATGCCCGAAAACTTGGTTGAGGCCGTACTCGAGGGCATAACA<br>CCGTTTCTGAACAAACACTCGGGAGTAGTCTTCGTGTCCTCGGTCA<br>TGGTATCTCGGGTGGGTAAATTCGGCGACGTCCCTACGAAGGCTG<br>ACGGCGGTACGTGATGAAGCCTCTGGTCAGAT
Thermo-crinis albus DabB	2317	GATCAAGGTCTCCCGTCCATGTTCCCAGAAGTGGCAATCGTTATCA<br>TACCGCTGTTAAGTATGATCACAAGCTTATTCACCGAGAAACGTAC<br>GTATGCCAAAGTGAGTACGCTCTTTACAGGCATGGCATTCTTACTG<br>TCATTATATGTTCTGATCTTCACGAACAAGGAGTCAAGTCTGTTATT<br>CTTACGCTTCGATGGCTTAGGGACGCTGCTGGCGTCATATATCTTA<br>TTGGTATCCACGGTCATCCACAAGTACTCTGAGAACTATATGAAGG<br>ATGAGCAGGGATTCAAGCGCTATTTTCTGCTGCTTGACTTAATGAC<br>ATGGAACTTACTCTTGCTCGTTTTGAGCAACCATCTGATTATTCTGT<br>TCGCGAGCTGGCATCTGATGGGAGTTATCCTCTATTTCCTGTTGAC<br>GTTTAACAATCGCCGGGAACAGGCCGTGCAAAGTGGCCGCACTGC<br>GCTTTTCACCCACCGCATCGCAGACGTACCGTTACTGGTTGCAATA<br>TTACTTCTGTATCAGCAATACGGTACGTTCGAGATCTCTAAGCTTGC<br>ACAAATGATTACCACCGGCCCGAGTGACACACTTTGGATTGTTACT<br>CTGCTGGTTATTCTTTCGGGAATCATTAAGTCAGCCCAAATTCCGTT<br>CCATGTATGGTTAGTGTATTCCATGGAAGGCCCGACGCCGGTAAG<br>CGCATTAATGCATGCTGGTATTGTTAATGCCGGGGCGTTCATCGCG<br>AATCGTTTCGCGTTCATGTTCCCCCATGATCTGTATGGTTTATCCCT<br>GTCTTTTCTGATCGGACTGATCACCGCAATTGTTGGTTCAACCTTGA<br>TGCTGATGCAAAATGACGTTAAGAAAGCTCTGGGTTATAGTACGGT<br>GGGGCAAATGGGCTACATGATGATGGAAATCGGGGTTGGCGCATT<br>CGCTCTGGCGCTGTATCATATGATGGCACATGGTATCTTTAAAGCG<br>ACCCTGTTTCTTAGCAGCGGTGGGGTGATCCATGAGGCCCGGCGC<br>GATACAAATATCCCTCGTGATGAGGTTTATGATGCGCTGGTGAAGC<br>GCGAGATGTCATTCAAGGAGATTCCTACGGTATTCTATGGTGCAGT<br>TACCCTGATTGTCCCCTTCGTTCTGGTGCTGGTAACCCATCTGTTC<br>TTCGAACAGGACGTGTTCCGCTATAAGGCCCCGCTGATACTGCTGT<br>TCTTCGGGTGGGTGACTAGCGCCCAAATTCTCTTCAATCTGTTTAA<br>GATGGGGAAGGAGAAGCCGCTGTTGACTATCTTTCTGGGTGGATTT<br>AGTCTGTTCTTACTGCTTTCCGTTTATACATTCATGAGTCATATATTA<br>CAAGTTTTCGTTTTCACCTATGAGGGGCTGCAAGAGGAGATTTATC<br>GGCGTGCATTCTCGAACGCCCCGTTATTCTTCCTGAGCATGATACT<br>GAGTCTTATTTTAGTGTTAGCCGGTTGGGTCCTGATTTATTTCGCTA<br>ATGAGGAGAAGCCTCTTAAGCTTCACCTTTCTCTGTATGCTCATTTG<br>TCGCGGGAGTTATATTTCCCAGACCTGTACAAGCTGACCGGCAAAT

		TATTCCTGCGTCTCGCAAGAGTGCTTTCTATTACATCATCTTCCGTT<br>GTTCCGGTGTATGGTTTCTTCTACCAAGGTGGTTCATCGGGATCTT<br>TCCTTCTCAAGGTTTTCCTCTTGAGTTTGTTCATACCGTTGTTTCCA<br>ATTTCCTTGATCACATCTTATTTGATCAAACGCTTTTGGATTTACTCA<br>TACCCGACCATTGCGTTATTAGGGTTCATTACTCTGAAACTGACTCA<br>CTTACCAGCGTACGAACCTCTGCACTACCTGGCCGCGCTCACTCT<br>GATTTTCCACAGTGTTCGTGCTACCCTGTCGGAGAATTTTAAAGAG<br>TCTGTCAGTGAGCTCTACCCGGCTCTGCTGTCAATAACATGGATCT<br>CAGGCGACGACCACTTCGTTCTGCTGCTCCTGCCGAGCCTGCTCT<br>TATACTTATTGGGAGTCTACATAAAGAAAGTTCTGCAGACAGATTCG<br>TTCTACTATGCGGGCGGGTTAATGGAGAAAATGCCGATATACAGCC<br>TGCTGCTCGTGATAGTCAGCCTGCAGGCATGTCTCACTCCCGTGAT<br>GCCGTCCTTCTATAGCTTCTTCGAGGCATTACTGCGTTCGAATACT<br>CTCCAGATTATACTCCTTGTTATGGGTTGGTTCACCTTGGGAGTTG<br>CCGTTGCATTATCAGTTTGGCGTTTGTTACACGGAAAACCACGAGA<br>CGATATACGTTATGCAGACATTCTACGGAGGTAACCCTATGCTCTG<br>GATCGAC
Therm-aero-bacter marian-ensis DabA	2670	CAGCTAGGTCTCATGAAGACCGCTACGACGCCACGGCAGCGGCCA<br>GGTCGCCCGGTTCATGGGCAAGGCGGCGGTCGACCAATGGTGGA<br>ACGTGTGCGTAGAGCATGTGATGCATTAGCACCGCTTTGGCCACTC<br>GCAACTTATATTGCTAGACACCCCTGGCCTGGTTTAGAGCGTATGC<br>CTTTTGCAGAAGCTTTGGATCATCTTCAACAAGTTCAAGGGGTAGA<br>CCTGCTTCCGCCCTTAGCGCTGTGTCGGGCGGCCCTCGCAAAAGG<br>TGAAATTGACCCAGCGGTTCTGGATCGTCGCTTGGCAAGATGGCT<br>GGATGACCATGTGTCACCGCAACTTAGACCTGAAGCTGAACGTGTT<br>TGTCGTGCTTTGCTGTGGCAAGAAGAAGTTCCGCGTCCTGAAGGT<br>GGGGTAAAAGCATGGGCCGCCTGGGTTGAGGAAGCGGCTCGTGC<br>GGGACGGTTTAGACATCGATCACAACGTGTTCTTCCCCGATCTGCC<br>CAACTTCGTGTTACAGCGCGCTTAGACGCCCAAATGATTCGTTGGT<br>GTAAATTGTTTCTGGACGAAGGGCAAGCCCGATGGCCATTACCATA<br>TCGTGAAGAGGGATTGTATCGCGCAGTTAGAAGACTTGTGCCATAT<br>GACCCTGCTTTGACGCGTGCAGAACGCCGACGTACAGCCGACTGG<br>CCGCTTGATGCCGAAGTGGCATTGGTTTGGGCTTTAGAGCGTCTG<br>GGTGTCGCGGACGCCGAGGTCGACGCTTATTTAGAAGCACATTTG<br>TTGGCTCTTCCAGGGTGGGCAGGTATGCTGTGGTGGCGGGGACG<br>CCAGGCTGGGGATGAAATGGCACCCCTTGTTGATTATCTTGCAGTC<br>CGTCTGGCCTTAGAATGGACTCTGTGTGCACCGGATCTGCCAGTG<br>ACAGGCCCAGCGGATCATCATGGGTCCGCAGTATTGCCATTGTTGT<br>GGGCATGGGAACGATGGGGTGGGATGACTCCGGCACGTTGGCGT<br>CGTTTGGATCCCGAGGAGCAAGAACGCCGTCTTGCGTTAGTCGAG<br>CGATTTCTGCGTATTGATCGTCGTCTTCTTTGGTTAGAAGCGTGGG<br>AAGAAACCTATGCAGCTCGACTGCGCCGCGCATTGACAGCTGGGA<br>GCCCGTCGGATGGAGATGCAAAACCCGCCCCCGCGGTCCAGTTGT<br>TGTTTTGTATAGACGTCCGTTCTGAGCCCCTTCGTCGGCATCTGGA<br>GCGTGCAGGCCCGTTTGAAACTTATGGATGCGCTGGTTTCTTTAAT<br>CTTCCAATTCGGAAACGTGAATTGGACTCGCCATACGCCCACCCGT<br>CTTGCCCCGCAATTGTAGAACCTCAGCATGAAATTGCAGAGCATGC<br>GGCCCCAGATGAATTAGCGCCTTTTAGACGTCGTCGGAATGTTCTG<br>CGGTTTGTGGGTCAATTATTTAAAACGTTAAAACAACATTTGTTAGC<br>TTCGCTGGTCCTGCCAGAGCTCTCTGGTCCCTGGTTGGGCCTGTA<br>CATGTTGGTTCAGTCTGCGTCTCCAGGGTGGGCAGGTCGTCTGTTT<br>CATAGAGCAGAGGCGGTAGTCAAACGTAAGCCACCAACTAGACTTA<br>CTCTTGAAAGAGTCGAAGCTGGTGGCAGTGCGGGGATACCAGTTG<br>GTATGCGTACCGAGGAAATGGTTCAAGCTGTTAAAAGTTTGTTCCT<br>TTCCATTGGACTTGTATCATTTGCACCACTTGTTGTTGTTTGTGGGC<br>ACCGTAGTTTATCTACGAATAATCCATACGCGGCAGCTTTAGAGTG

		TGGTGCTTGTGGTGGTGCAGCCGGTGGTTTTAATGCCCGTGTGTTT<br>GCGGCTCTTTGTAACCGGCGTGATGTTAGAGAAGGTTTGGCCCGC<br>GAGGGTTTGCGTATACCAGATGAAACCGTTTTCGTGGCTGCAGAAC<br>ATGTAACTACCTTAGACGTTCTGCAATGGGTAGATGTCCCGCCTCT<br>TACGCCTGCAGCGCAAGAAGCTTTCGCGCGTTTATTACCGGTTTTG<br>GATCAAGTTTCTCGGCGGACACGCGCAGAACGTGTTGTAAAGCTTC<br>CTCATGTTGGGGCCGTACGGGACCCACATGCGGAAGCGTGTCGTA<br>GAGCGACCGATTGGAGTGAAGTCAGACCAGAGTGGGGCCTCGCG<br>GGAAACGCGGCGTTCGTAGTAGGAAGACGAGCCTTAACGCGTCAT<br>GTGCACCTTGATGGCCGAGTATTTCTTCATTCGTATGATTGGCGAT<br>CGGATCCATATGGTGAACGTTTAGCCGCTATAGTTGCAGGTCCTGT<br>TACTGTTGGACAGTGGATAAATTTACAATATTATGCATCGACTGTAG<br>CTCCCCACGTTTACGGATCAGGTAGTAAAGCCACACAAACTGTTAC<br>AGCAGGTATTGGGGTCATGCAAGGAAATGGATCGGATCTCATGACT<br>GGTCTTCCATGGCAATCAGTTGCAGCATCTGATCGCGAGGTAGTG<br>CATGCGCCTTTGCGTCTTCTTGTAGTGATAGAAGCCCCGCGTGAGT<br>GGATACAAAGACTTTTGCAACGTGATGCCCAATTTAGACAAAAGGT<br>TCGCCATGGTTGGATAAGACTTGCTTCTGTAGATCCAGTACGGGGC<br>GAATGGATGGACTGGGAACCCTCAGCAGTTGATGTCGCACTGTCA<br>CGTGCGGGCTGAAGCCTCTGGTCAGAT
Therm-aero-bacter marian-ensis DabB	1541	GATCAAGGTCTCCCGTCCATGGGGTTGATGGCATGGTGGACATTA<br>GCAGGTAGTTGGGGCTTGGCCGTTGCCTCTGCAGCCGTCTGGAAC<br>TTACGTAGAATGCCCGAAGGTTACGGGCGTTTGCACGTTGGGATTA<br>TGCTGCTGCCTATAGCGGCAGCCTTGTGGGGTCTTCGAACTGCTC<br>CCGCGGTGATAGAAGTCGGACCGTGGCGTGTGGACGCTTTGGGG<br>TGGATGATAGCACTTTATGTTGCTGCTCTGAGTGCAGCAATTCAAC<br>GTTTTACTCTCCGGTATTTGCTTGGCGATAGATCGTATAGACAAGTC<br>TTTCTGTTGTTAACGTTAACTACAGGAGCGGCGGCTTTTACTTGGG<br>TATCTGATGATGTTCGATTATTTGTTTTGAGCTGGACTCTTATGGGA<br>GCGGGTTTGGTCGCTCTCGTTGCACTTAAACGCGAATGGATGCCT<br>GCTAAAGTAGTTGCATTAACAATGGCGAAGGTTTTCGCACTGTCCG<br>CCCTCGCTTGCGCAGTTGCAATGGGGTGGCTCGCTTGGGCTACTG<br>GCTCATGGCGTCTGAGCGAAGCAATGGCACATGCTGCCGCCTTGG<br>GTCGAGGACATCAAGTGGGCATAGGTGTATTACTGGTAGTCGCAG<br>CTCTGCTTCATGCAGGCCAGTGGCCTTTTCATCGCTGGTTATTAGA<br>ATCCGCAGTATCTCCTACGCCAGTCTCAGCTGTAATGCATGCTGGT<br>ATAGTGAACGCGGGTGGCTTGCTGTTAGCACGCAGTGCTCCTTTGT<br>TAGACGCTGCTGGTTCAGGTGTAGCGGTCGCGCTGCTGACAGTCG<br>CAGGTTTGTCTGCACTGTTAGGCACAGCTATTAGCTTGGTACATGT<br>TGATTATAAAAGACAACTCGTTGCTTCAACAATGGCTCAGATGGGT<br>CTGATGTTAGTGCAGGCAGCGTTAGGGGCTTATGAAGCAGCTATTG<br>CTCATTTAGTCTTACATGGTTTGTTTAAAGCCACACTTTTCCTCAGA<br>GCTGGTTCTGCAGTCCCACGTCCAGAGGAACGCGTTTATGCCCCC<br>GGCTCTGGAAGACCCGTCCACCCAGCGGTTGGAGCCTTATTTGGT<br>GGATTAGCCGGTGGCGCTTTCTGGGCTGAAGCAAGCGGGGAACCA<br>GCTCGCTTACTGTCAGCCCTGTTCTTCGGGGCAGCGGTAGCTTTA<br>GCATGGGGTCAAGTTCCGATTTATCGCGAGGGTCGCTGGCTCGGT<br>GTAGCAGCCGCCGCACTGATGGCTTTAGCCAGCGAAGGCGTACGT<br>GTTACATTAGCAGAAGGCGTGCGTGTCGCTCTGCCCCCGGGGACA<br>AGCCCATCTCCTGTTGTTGAGGGACTGGCTGCCGTTTTATTCGCGA<br>CGGGCGCAGGTCTTCTCGCCCTTGTAGCCCGTCGGCGCACTACGG<br>GCGCTTTCGTTAGACTCTATATGACTTTGGTTAGTTTAGGCGAACCT<br>AGACCTGCTGCTATGGATCGTCACCCGCGCTATCTTGCCGCTTACG<br>TGAAGGAGGCGATTTTGCGATGAAGCTCTGGATCGAC
Alicyclo-bacillus	2574	CAGCTAGGTCTCGTGACGCGCTCCCGTCTCGCTCCCGCTGGAACC<br>ACGTGGACGGCGTCGTCCGCTGTTTCTGCAGCGCGTCTGGCTCTG

acido-caldarius DabA	GATGTAACAGCAGTGCAATGGCCTATATCTGTTTTCATTGCGCGTC ATCCATGGCCTGCCCTGGAAGATCTTCCATTCGCCGCGGCCATGC GTAAGCTTCAAGCCCTGGGCGGTGCGCACTTATACCCGTCTATGC GTCTTTTACGTGATGCACTTCACCGTGGAGAAATCGACCCTAACAG ATTGCGTCACAGAATGGACCAATATCTCGAGGACGATGTCCCAGCC CACTTGCGGGGCGCTTTTGCGTCAGTCCAGGAAGCACTGACTCAC GAGGCTGGGGACCCAAGTGATGCATCACCGGAAGCGATGGCTCTG GCAGCAAAAGCTGCGGCAGCACCTGACTTGCCGGATGAAGCATTG CATTTCCCTCTGCCATCGGATGGAGAACGTGCACGTGTTGATGCCT TGAGTATACGTTACTTAAAACTGTTCTTGGATAGAGGACAAGCAGC TTGGAGCATGCCTCTGCGGAGAGAAGGCTTATTCCGCGCAGCACG TGCTCTGGCAGGCCGTGACCCTAGTCTTAGTCGTGCTGAGAAACG CCGTCTCGGGGAGTTACCTGACGCTTCTGACGAAGTTTTAGCATTC GGATTGGAACGGTTCGATGTTAGCCCCAGATTTGCAGCCGATTATT TTCGAGTACACTATCTGCGCCTGCCCGGTTTTGTAGGAGCTCTCAG ATTTCTGGGCCGCGAACAAGGTCTTGAGGACCGTTGGATGCTGGA TTATCTCGCCATGCGGATTATGATTGAATGGGCACTTGCCGGAGAC GCCGGTAGAACCTTCCTGCCACATCCAGATCTGGCAGGGTTGGCG ACCTCGGCAGCAAGATTGTTGGAAGCTGCACGTGGACCGGCTTCA GTGGACCTGCTGCTGCTGGCTCACAGATATCGTGTCGCAGACCGC TATGCCGTCTGGCTTGATGCTTGGGAAGAAACCCATGAAGCACGG GTTGTACGTCGTGGTTTGGCCTTGTCAAGAGAAAATACCGTCCCTA AAGCACAATTTCTTTTCTGTATTGATGTTCGTAGTGAACCCTTACGT CGTCATCTTGAAGCAGAAGGCCCATATGAAACCTTTGGCTGTGCCG GCTTCTTTAACTTACCCGTATGGACACGTTCTTTAGATAGCTCATAT GCACATCCCTCGTGCCCGGCTATTGTACGCCCTGTAGCCGAAGTC TGTGAAGAAGCGGTTGATGAAGCCGGGCTTGCAAGATCTCGCCGG TTAATGGGAGCATGGCGCACCCTGGCCCAATCGTTTAAGAAGGTG AAACAATCCGGTGCGGCAAGCTTAGCGCTGCCTGAATTGAGTGGA CCCTGGCTTGCTTTAGATGCCGTAGCTCGTACAGTGCCCCCGTTAC GGGCGTTTGCAGCGCGAGTTGCCAGAGCAACGCTTCCACAAGCCG AAACTCGTCTGGCTTTAGACCGTCGGGAAGGCCCCGCAGGCGTTC CTCTCGGACTGAGTTCTGAAGAAATGGCAAAATTTGCAGCCGATCT TATCAGAAGTATAGGACTTACACATTTCGCCCCATTTGTAGTCGTTT GTGGCCATGAAGCCCGAGTTGAGAATAATGCCCATCGGGCAGCTC TCGATTGTGGTGCATGTGGTGGCCGCAGCGGTCGTACAAATGCCC GTGCACTGGCCGCAGTACTGAATCGGGCAGATGTAAGACGTCGTC TTGCAGCAGAGCACGGTATATTTATCCCTGGGTCAACACGCTTCTT AGCGGCTGTCCATGTAACGACGACAGATGAAATTGAATGGTTGGAT GTACCACCACTGGTAGGAGAAGCACGGGTGCAATTCGAAGCACTC TCGGCGGCAGTCCGTCGTGCGGGCGAGAAAGCGGCATCAGAACG CGCCCAAGCGTTACCTGGTGCTAAGCGCGCACGTCCAACCTTAGA AGTTCGCCGACGGGCCTCTGATTGGGCAGAAGTACGACCCGAATG GGGCCTGGCCCGAAATCGGGCATTCTGGATTGGTCGTTTGGCACT GGACAGTGGCCCGGTAGCTGGTGAAGCCTTCTTACATAGTTATGAT TGGCGTCTCGACCCTGATATACGTGGCTTAAAATCTATCGTTGCGG GACCTGTCACCGTAGCACAATGGATAAATTTACAATACTACGCCAG TACAGTAGCACCGCATGCACACGGTGCAGGTTCCAAACCTACACAA ACCGCCACTTCGGGGATTGGAGTTATGCAAGGTAATGCCAGTGAC TTACTTCCTGGTTTACCTTGGCAATCAGTTGCTAGTGATGATGCGC ATCTGTATCACAGACCGATACGTCTCCTTGTGATTATTGAAGCACCA CGCCGTATGGTAGAAAGATTGTTGCGTGAGGATGAAGGCTTCCGT CGGAAAGTCGAAAACGGCTGGCTCCGTCTGCTTATATTTGACCCAG AGCGCGGGGCATGGGCTCGTGGAGAGGCAGTAATCCGTACAAGC GCGAGAGGTTGAAGCCTCTGGTCAGAT

Alicyclo-bacillus acido-caldarius DabB	1550	GATCAAGGTCTCCCGTCCATGGACTTTGCTGCCGTCCATCAGGCC<br>GTTTCACTCGTATGGATGTTAGCTTGGATTGTTGCGTCAATGACAG<br>GAGCGCTGCTGTGGCCATTGAGAGAACCGAGCAGACGAATTCGCA<br>TGCATTTAGCTTCCCTGTGTCTTGTTAGTTTAGTTAGTATGTTGGGA<br>TTGGTCACAGGGGCAATTGGGGTTCGATTAGGCCCTTTACATCCGA<br>CGGGATTGGGTTGGGCAATTAGCTCATATATTTCTGCACTTGGATT<br>AGTGGTTCAAACCTTTTCAGCCCGGCATTTAGCAGGTGATGAACGA<br>TATGGTTCTTACTTCGCCTGGATGACCTGGACGCTGTTCTTCGCGT<br>CCGCTGCTTGGATGGCAGGGGACGCATGGTTCTTTGCAGCATGCT<br>GGGTTGGCATGGACGCTGGTCTTATCAGACTCATCGCTATGCAAC<br>GCCGTAGTCGCGCAGCCCAGGCAGTTGCTCGCATGACTTGGGCTC<br>GTTTGGCTCCATCGATGGCAGGTGTTTTACTGTTATGTGGGATGGC<br>CGCATGGGCAGGACGTAGCCCTTCACTGGACACCGGAATACGTGC<br>TCTTGCGGCTCATCCGCATGCTAGTTTGGTCGCGGGGTTATTACTG<br>GGTACAGTCGCACTGGCACAAGCCGGTAATTGGCCGTTTGGTCGG<br>TGGCTTTTGGATTCTGCAGTGACTCCTACCCCAGTGTCTGCGCTGA<br>TGCATGCTGGGTTAGTTAATGCTGGTGGGCTTTTACTGGCAAAATT<br>CGCACCTGTGCTGGCCGCTGCAGGCCCTTTCCCAAGAGCTCTGTT<br>ATTGGCTTTTGCATGGCTGTCAGTTGTGACGGGTACGGGTGCTATA<br>TTAGTCCAAGCAGATTATAAACGGCAACTGGTTGCCTCAACGATGG<br>CACAAATGGGACTCATGTTAATGGAATGTGCAATGGGTGCATATGC<br>CGTTGCTATAGTCCATCTCGTTTTGCATGGTATCTTTAAAGCATCCT<br>TATTTCTTAAGTCAGGCTACGCTGTACCTGCTCCTGAAGATGCACT<br>GGTCCCACCAAGTCCTCAACCTAAAAGATCTCTCTGGCCTGCGGTC<br>GCGGGAACGCTGTTCTTACTCGCTTACGTTGCACCCCATCCTGCG<br>GACGGGATGCGTCTGCTGAGCGGACTGTTACTGGCAGCTGGGTGT<br>GCCATTGCCGTCAGATTGGCAGCTGATCTCCAAGCAGGTCGTTGG<br>CTTGGACTGGCAGCGGTAGCAGCCGCAGCTGGTGCTGCTGAGGG<br>CGTGCGGGCAGCATTAGTACGGGCAATAGCAAGTCTGGGTGCATC<br>GAATGCGCATCCGGATTGGGCTTTTGCGGTATTAGCAGCTGCTTTA<br>GTTGCATTACAAGCCTTTGCTTTTGCCTGGTGGCGTTGGCGCCCAG<br>ATACCAATGGTGCTATGCTGGCGTATGCTTGGTTGGTCCACTTGAG<br>CGACCCCCGACCAGCCTCGGTAGAGGCGCAGCCCACGTCCCTGA<br>AACAACTGGTGAAGGAGGCGATTTTGAAGTGACGCTCTGGATCGA<br>C
Fonti-monas thermos-phila DabA	2598	ATCCAGGGTCTCATGCAGACCTCGCCGTCCTCGCCGGCCCCCGTT<br>CACGGCGCTCATTCCTGCCGATCCGCGGCGGCTGCGGCCACACC<br>GGATTCGGGCGATGTGCGGGGTATGGATCTATATCAGCGCATCGA<br>AGCAGCCTGCGAGCGGGCGTGTCAGCAGATCGCGCCGGTCTGGC<br>CGCTGGATCGCGCAATCGCCGTCAATCCACACTGGTGCCGCATCG<br>ATCGATCCGTGCGCACGGTTGCCGCACGTATGGCTGTTCTCGGTC<br>ACATCCAGGTGTTTCCACCCCGCGACATGGTGCGGCAGGCCTGGG<br>AAGAAGGCCGCATCACTGCGGCGGATCTGGCTTACGCGCTCGATA<br>CGCTACCCGCAGCGCAAACAGCCGGTCTGACTCCGTCGGGCTGC<br>GTGCAGGCTCTGGCGGCAGCGCCGGCCCTTCCGCATTTGCCGCT<br>CTTGATCGATGTGCTCGACGACGACCCGCTGCGCCACACACGGCT<br>GTCCTGGCGGCAGGCGATCACCCATCAGGTCAGCCAGACCTGTGC<br>GGCCTACTTCGATGAGCACCAAGCCGATTGGCACCCGGACCGCCG<br>GCACGGTTTGTACGCGTTCTGGCGCGATACCCTGACGCACGACCA<br>CGGTATCGGCGTGCTGATGGGCTTGCCGCATCTCGGACGCAGCCT<br>GCATGCCCTGCCCGCCACGCGGCAGGAGGCGGAGTCTTGGGTCT<br>TGCAGCGGCTGGGTCTGCCTGAATCCGTGTGGGCGGATTACTTGG<br>AGGCAGTTCTCCTCACAGTTAATGGTTGGGCCTCATGGTGTGCGTA<br>TTTAGATTGGCAAGCACGTCAAGCAGGTCAAAGAGATATGCACCTG<br>CGCGAACTGCTGGCTATACGATTAGCGTGGGGAGCAATATTACTC<br>GAGTGTAAAGAGGACGCCTCTGCTAGACGTGCCTTCGCTGCGGTT

		CAAGCCCAGTGGAATCGGGCTGATACCTTGCTTCAGAAGGCCGAA<br>GCTTTACTTCTTGTTGATGAGGTTTGGCAACTCGCTTTTGAAGCCG<br>GCTACCAACGCCGCCTCGCTCGTCAATTACGTACCGCAGCTACAC<br>CTGCCGCACCACAACAAGTAGAAGTCCAAGCTGTGTTCTGTATAGA<br>TGTCAGATCTGAGCCGGTGCGGAGAGCTCTCGAAGCGTGCTCTCC<br>AAGCATCCAAACTATTGGTTTCGCTGGTTTCTTCGGGCTCCCGGCA<br>GCGTATACTCCTTTAGCTACTCCAGCCCGGCGGCCTCAGTTACCTG<br>GTCTGTTAGCTCCGAGTGTTGAGGTTGTAGACCGCGTTGTTGCGG<br>CTCCGGGTCAATCAGGTGCACCGACACCAGACTTAACTGTCGCCG<br>CTACACATGCCCGACAACGTCGCTTTGCCTGGGGCGGCCCATGGC<br>GCGCGGCTTCTAGATGGCCGTCAGCAGCCTATTCGTTTGTAGAGG<br>CAGTAGGTGTAGGCTACCTGGGACCTCTTATGCGTTGGCTCTGGC<br>CGTCCCCCGCGGCTTGGACACATGAAGACCACTATGGACTGCCTG<br>AACGATATCGCACAATTTGTCGACCTACGTTAATTGGCCTGGACGT<br>TGAAGAAAAGGTGGAACTTGCCGCTCGCGTATTACATGCCATGGG<br>ACTGGACCGGCGGGTAGCCCCATTAGTGCTGTTGGTTGGCCATGG<br>ATCGCAATCTGTTAATAATGCACACGCAGCAACCCTTGACTGTGGG<br>GCGTGCTGCGGTCAGACCGGGGAAGTGAATGCGCGTGCACTGGC<br>TCAGCTTTTGAACGAACCGGCAGTTAGAGCGGGGCTGCGTGATAA<br>AGGCATTGTAATACCGGAAGATACTCAATTTGTAGCAGTTCTTCACA<br>ATACTACAACCGACGAAATAGAGGGATTTGACCTGGATCGCCTTCC<br>CCCAGCTGCTCGAGCTCGTTGGACCCAACTGCGGCCTGTTTTGGC<br>ACAAGCGGGCGATCGTGTAAGACGCGAACGCGCCCCTAGACTTGG<br>ACTGGATGCCCAAGCGGACGCTAGACAGCTGCTGGCCCATATGCG<br>CCAACGTGCTTCAGACGGAGCTCAAACTCGACCAGAATGGGGATT<br>AGCGGGTAACGCGGCATTCTTAATTGCTCCAAGAAGCCGTTCCCG<br>CCACTTAATGCTTGATGGGCGGTGTTTCCTGCATGATTATGATCCT<br>GCGAATGACCCAGATGGGTCAGTCCTTGAATTATTATTGACTGCAC<br>CTATGTTAGTTACTCATTGGATTAATTGGCAATATCATGCAAGTCTG<br>TGTGACCCTCAGCGCCTTGGCTCTGGAAATAAGGTTCTTCATAATG<br>TAGTAGGCGGTCATATTGGCGTTTTCGAGGGTAACGGTGGCGATTT<br>GCGAATTGGCCTCTCTAAGCAAAGTCTTCATGATGGGACTCGCTGG<br>GTACATGAACCGATCCGTTTAACTGTCGTCGTGGACGCACCGGCG<br>GCGGCAATTGAGGGTGTAATCGCTCGGCACGCTGTCGTACGACAA<br>CTTCTGGATAATGGATGGCTGCACCTTTGGCGGTATCAAGATGCTG<br>AGTTGCTTCGTTACGCAAACGGCCAATGGCAAGTCCTGGAATTAGA<br>ATGAAGCCTCTGGTCAGAT
Fonti-monas thermos-phila DabB	1607	GATCAAGGTCTCCCGTCCATGCTGGCCAGTGCTTTTATGCCCGCAC<br>TGGCAATGACGCTGGCCGGGTTGCTGGTATGCCGTTCGTCCGCTG<br>GCGCCAGAGCGCTGTGGCGGTGCTTTGCATGGTTGGCAGGTGTG<br>GCATTGGCGGGCGCTGTCGTGCTCCTCGGATGGCAATGGGGTCTG<br>CCGACGCAGGCTATCGATCGCGGCGCAGCATCGGCTTGGTTTGCA<br>ACCAGCCCCCTTACCGCTTCGCTGGCAGTGCTCGTGCAGTTTCTC<br>GGCGTAGTCATCGGGGCGTTTTCGTCCCGGTATCTGGAGGGCGAG<br>GCGGGCCAGCGGCGCTATGTCGCCGCGCTTTGCGGAGTGCTCGC<br>TGCCGTACAACTGCTTTTGCTCGCCAACCACTGGCTGCCGCTGATC<br>GCGGCCTGGGTCGCCGTGGGGGCGGCCTTGCAGCCGTTGCTGTG<br>TTTTTACCCGGAACGGCCGTTCGCGTTGTTGGCAGCGCACAAGAA<br>ACGTCTGGCCGACCGGCTGGCCGACCTGTTGCTGATCGGCGCCG<br>CCGGTCTTGCCTGGTGGTCTGCAGGGAGTGGCTCTTTCGATGATC<br>TTTACGCACACCTCGCCGACGCGCAGGCTTCGGCCCTGCTGTCAT<br>GGAGCGCGGTGCTGCTTGCGGGGGCGGTGATCCTGCGTACGGCC<br>CTACTGCCGGTTCACGGCTGGTTGATTCAGGTGATGGAAGCGCCA<br>ACCCCGGTATCCGCGCTATTGCATGCCGGGGTGGTCAACCTCGGT<br>GGGATCGTGTTGATCCGGCTGGCGCCGCTGGTGGAAGCGTCGAC<br>AGCGGCGCGCTGGATATTGGTCGTCTTCGGTCTGGGGACGGCGAT

		GCTCGCCGGTCTGGTGATGCTGACGCGCATCAGCATCAAGGTCAG<br>GCTTGCGTGGTCTACCGTGGCCCAGATGGGCTTCATGGTCTTGGA<br>GTGCGGGTTGGGTCTATACACGCTGGCGGCGTTGCACCTCATCGG<br>TCACTCGCTCTACAAGGCCCATACCTTCCTCGCTGCTTCGACCGTG<br>GTGCGCCAGAGTCGCCAGCGCATGATGCGCGCAGAGGCGCGAGC<br>GTCGTTGCCCAGCCTGCTGGCGGCACCGTGGGTGGCGCTGGCGA<br>TGGTTGCAGCAGCGCAAGCCCCCTTTGCGGCATCCGCTTGGCCGC<br>TGTGGTGGAGTGGCGTTCTGGCTTTGGCATGGTCGCCTTTGCTGT<br>GGCTGCCCGCGGCGCAGGCCAATGTTCCAGGTGTGTCCCTCTTTC<br>GTGTCCTCGGGGGACTGGCTCTGATCGGTGGACTGACGGCTCTGG<br>CGCAGTTGCTGCACGGGCTGCCGCTGGGGCTTGAGGATCGGTCA<br>GCGGATGCTGCCGGCATGGTTACTTTGCTCGGCATGGTAGCGATG<br>TACCTGGCATTGCTTACCTTGCAGTGGCGGCCGCGCTGGCTCGAG<br>GGCGCGCGGCGCTGGAGCTATGCCGGGTTTTATGTGGATGAGTTT<br>TATACGCGCCTGGCGTTGCGATTTTGGCCCGCCGCCTGGACACCG<br>GCGGCAGCATCAGCGGCTGCAGCACCGTCCGGTTCGCGTCAGTAA<br>CCGTTGCGTCGGAGCATTCTATGCAGCTCTGGATCGAC
Acidithio-bacillus caldus DabB	1679	GATCAAGGTCTCCCGTCCGTTATGCCTTCTGTCCTCGTGCTTAGCG<br>CTCTGCCCTCTGTACGTCCTACGCGTCAATCTGCGCTGGGCACTC<br>GGTACCGCGTCCGCTGGGCGGCTATCGTAGCATTGTTAGGCGCTC<br>TTGCGGGAGATATTGGATATTTTCTGGGGAGCCAAACCTCGTTGGA<br>AATTTTCAAAATTACATTGCCTGGCTTAGGGTTTGCTCTGCCATTAA<br>GCGTGGCAGTGAACGGCCTGACAATGGTGTTAGCAACGCTTGTGT<br>CATTTGTCATTGTCATGATCACACAATATTCCGTTGAGTACCTTGAC<br>GGTGACCCCCACCAAGCTCGTTTCTTTCGGTTATTAGCGTTCACCG<br>GCGGTTTCTTCTTACTCGTGGTAGTCAGCGGCAATATTGGGCTTTT<br>CACACTTGGCATCATTGCCACCGGCTTTTCACTGAATAAATTATTAA<br>AATTTTATAATACTCACCCTAAAGCCATAATGGCCGCGCATAAGAAG<br>AGCATCTTCACCAGAACGGCCGACTTATTTCTGGTTGGTGCTAGTG<br>CCTTGATAGGCTCGCATGTTGGTTCTCTGCAGTTTGACGCATTACG<br>TCAATTCGTGGCTCACGCAACCGAAATTCCTTTCGCCCTGCAAATA<br>GCTGCTTGGCTTATTATAGGCGCAGTGATTTTAAAGAGCGCACATT<br>TCCCGTTTCAGGGTTGGTTAATCCAAGTCATGGAAGCGCCGACCC<br>CCGTAAGTGCTCTCATGCACGCAGGGGTGGTGTACAGTGGCGCAA<br>TCATCGCTTTAAGAACTACGTCCTTACTGGTTCGCGTATCCGACGC<br>ACTCCTCTTCTTAGGCATCATGGGCCTTGCAACAGTGGTAATTGCC<br>AGTCTGGCGATGACGACTCAAACCGCCGTTAAATCTATGCTCGCAT<br>GGAGCACCACCGCACAACTCGGATTTATGTCCCTTGAGCTGGGTTT<br>AGGTTTATTTCCACTGGCCCTGTTGCATCTCATCGGGCATAGCTTA<br>TATAAAGCTCATGCGTTCTTAAGCTCTGGGTCCGTTCCGGATCAAT<br>TACGTCAGGTTCCCCCGGGTGGCAAGAAGATACCGAGCATGACCT<br>CATGGGTGATGGCTGTAGTGTTAGGCTTAATTATTTCCGGCGGCGG<br>CGCTTGGGTATTTGGTGACGACCCTCTGAAGGACCCCACATGGAT<br>GGCGTTGGTTGTAATTATAGCGGTCGCAGTGTCTCAGATTCTGATT<br>AAGGGATTCCAGTTTGACGCTATCATTGATCGCTTCGTCGCCTTCT<br>TAGTGGCCGTCTCAATGGGCTTCACTTACTTGATTTTGCATACCCT<br>GTTCGTTTGGGGATTTACGCGCGACATGACAGAAGCCATGATGCC<br>GATTACGACACCGTACCTTTGGCTGTTGGGCTTGAGCATGGCTAGT<br>TTCTTAATTCTGAGCTGGTTACAAGGACCAGGCCGAGCGCTGTTAC<br>CCCAGAAGATACAGCTGGTCTTGTTTACTCACCTGTATAATGGTCTT<br>TACGTAGACGTTTGGGTTGAACGCATCTCCCATCGATTTTGGAGCG<br>AGCGTGTCGGTGTGGAGTTACCACGGAAGAACTTGGCCGTCGAGT<br>CACTGCAGTCCATTGCCCGAAACCAAGACGCATTTGGTCAATCTGG<br>AGGTGTACAATGAGCCTCTGGATCGAC
Acidithio-bacillus	2433	ACTGACGGTCTCATGAGCTCTAACAAGACGGGAAACACAGAACGG<br>TTGTTTGATCGCGTCATTCGACGTATTGCCCCGGTGTGGCCATTAG

caldus<br>DabA	ATTCCTTCGTCGCTGTGAACCCGTATTGGGGCTTCGCAGATCAAAC<br>ATTCGCAGAAGCTGCCGCACATCTCCAGGGTAGTGTTGGAGAACG<br>TGTGATTATGGACAGACGGTGGTACGCAGACCTGTTAAAGAATGGT<br>AAATTGATGATGGCAGATATCCTTCAAGCAAGTAGTGATCTGGGTG<br>TGGACTTAGACGAATACGGATGGCGTGCATACCTTAGAGAGCCGT<br>CCGAACTGCCCATGAGACTGCCCCGTATTCCTGAGCTGATGGATC<br>GACCAGGTCACCCAGCCGTGTCTCAGTATGTTGTAGAACAAATTTC<br>GCGTTTTCTGGCGGCCTATTATGACCGCGGTCAATCACTGTGGAGT<br>TTTCCTAAAGACCCCGCCGTTGGTCTGTTCGGGAGCTGGAGACAAT<br>ATACTCTGCTGGACCGTACTCTTCGGGCTATGGGGTTAAAACATCT<br>GCGTTCAGAACTTTTGACGGTCAGTAGCAACGCCGCATCTGCGCG<br>CCAGTGGGCGCTGGAAACCCTGGCAATAGCACAACCCGCGGAAGA<br>GGATTATCTGTTAGTGCTCTTGAAGTCGATTGGCGGCTGGGCGTCA<br>TGGTGTCGGTATCTTCTTTGGCAAGCTGAATTGCAAGGCTCTACCA<br>ACGCGGATTTGTTAGACCTGTTAACTATCCGTATGGTATGGGAAGG<br>TTTGTTACGCAAAACTGCGGATGCACGTGTCCTCGAAAGATGGAGA<br>CGTCAGATACATGCGTGGACCATTGATGATAAAGGTGTTGATTTAG<br>AGGACGCTAGACGCGGCGAGGTTCTGCTGAGAGCGAGTGAAATCG<br>CATATCGACGTCAAGTGGCGGCCTCAATCCGGCGGCAGCCCGCTA<br>AGAAGAACCGTGCCCCGTTAATCCAAGCGGCGTTCTGTATTGATGT<br>GAGAAGCGAAGTTTTCCGAAGACACTTGGAGTCAGTCATGCCCGA<br>ATTGGATACAATAGGTTTCGCAGGCTTCTTTGGTGTACTGCTCGAC<br>TATCAACGTAACGGGGACTACGCACCACGAACACAGACGCCAGTA<br>TTACTGAACCCACAAGTTCATGTTACGGAAGACGCTCCAGGTGATG<br>CCATACAAAGACGGTTCCGCCGTCTTCGTCGCGCAGCGGAATGGA<br>AACATTTTAAACTGTCAGCCGCGTCATGCTTCTCGTTTGTCGAGAC<br>AGCCGGGATAACGTATGTGGGACGGCTTTTGGCCGATACAATGGG<br>CTGGCACCGGCCTAGCGTACACCCCGATGAAGCTGGTTTGTCACC<br>GGAAGAACGTGCAAATCTTCGCTGTGTGCTGCCTAAGAGTTTGACG<br>CTGGAACAAAGAATCGGTATGGCTGAATTTATCTTGACAGGTCTGG<br>GATTAAATCGTGGGGTTGCACCAATCGTATTACTGGCGGGTCATGG<br>CAGTAGCAATACTAACAACCCACACCGTGCGGGATTGGACTGTGG<br>AGCTTGCGCCGGACAAACTGGGGAAGTTAATGCGAAAGCAGCCGC<br>GGATTTATTAAATGACCCGGCAGTCCGCAAGGGTCTGGTCGAAAA<br>GGGTTGGAACATTGACCCGCGTTGTTGTTTCTTGCCGGCATTACAT<br>GATACAACTACTGACCGCGTAGAAATCTTAGGCGGCTTGGACAATC<br>CAAAACTGGATACTGGCTTAGTGGCGGAACTCCAAGCCGCCCTTG<br>ACAAGGCTGCAAATCTTACTCGCTTGGAGCGTATGCTGAGACTGGA<br>ACCAGATCTCCGGGACCCAGAAACTGTGGCCCGTAATATGGAACA<br>ACGTGGACGTGATTGGAGTCAGGTGCGCCCGGAGTGGGCCCTGG<br>CGGGAAACGCAGCCTTTATAGCTGCGCCACGTTGGCGTACACGTG<br>AAATGAACCTGGCCGGCCGTGCGTTTCTCCATGATTATGATATAGA<br>AAAGGACCCCGACTTTGGGGTCCTTACATTAATTATGACAGCCCCA<br>TTGGTCGTTGCTAATTGGATAAATCTGCAATACTATGGATCAATTGT<br>AGATAATCAGAGACAGGGGTGTGGAAATAAGGTGTTACATAATGTA<br>GTAGGTGGTACAATTGGTGTCTTGGAAGGCAACGGTGGTGACTTG<br>CGCATCGGTTTAAGCGAGCAGTCACTGCGTGATTCTAACTCAGAGT<br>TGCAACATGAGCCGCTTCGTCTGACAGCGTTTATCGAAGCTCCTAC<br>GGATGCGATGGACCGTATAATAGCCAATAATGATGCCTTAGGGAGA<br>CTGGTGAATAATCGCTGGTTATCAGTAGTTCAAATAGCTGCGGACG<br>GTAGTCTTCGCGAACGTCGCTCCGAAGGACGGTGGGTATCAATTT<br>GAAGCCTCTGGTCAGAT

Table S6. Curated List of Diverse Uniprot DAB1 Sequences used for Conservation Analysis.

DabA Entry	Gene Names	Organism	Associated DabB Entry	DabC present?
A0A059ZT80	dabA Acaty_c0924	*Acidithiobacillus caldus* (strain ATCC 51756 / DSM 8584 / KU)	A0A059ZTL4	yes
A0A060UT06	dabA AFERRI_20539 AFERRI_370022	*Acidithiobacillus ferrivorans*	A0A060UN95	yes
A0A0K6HSN6	dabA Ga0061069_101421	*Thiomonas bhubaneswarensis*	A0A0K6HSR8	yes
A0A0P9C3F5	dabA SAMN05661077_0175	*Thiohalorhabdus denitrificans*	A0A1G5AAE3	yes
A0A179BQF4	dabA A4H96_00055	*Acidithiobacillus ferrooxidans (Thiobacillus ferrooxidans)*	A0A179BQN6	yes
A0A191ZGR6	dabA A9404_06355	*Halothiobacillus diazotrophicus*	A0A191ZGS1	yes
A0ACD5HCP 7	DUF2309 domain-containing protein, HHS34_009510	*Acidithiobacillus montserratensis* (strain GG1-14)	A0ACD5HD5 5	yes
A0A1D8IK76	dabA BI364_01595	*Acidihalobacter yilgarnensis*	A0A1D8IK93	yes
A0A1D8KAU1	dabA BJI67_14225	*Acidihalobacter aeolianus*	A0A1D8KAS2	yes
A0A1H3ZAL0	dabA SAMN05660964_0111 2	*Thiothrix caldifontis*	A0A1H3ZAN1	yes
A0A1Z4VPS3	dabA FOKN1_1083	*Thiohalobacter thiocyanaticus*	A0A1Z4VPR4	yes
A0AAE3CKJ7	dabA HFQ13_12175	*Igneacidithiobacillus copahuensis* (strain VAN18-1)	A0AAE2YRL6	yes
A0A285P3L8	dabA SAMN06265353_1106	*Hydrogenobacter hydrogenophilus*	A0A285NYE1	yes--fused
A0A2I1DKH3	dabA B1757_09710	*Acidithiobacillus marinus*	A0A2I1DKG2	yes
A0A368HBX8	dabA C4900_08585	*Acidiferrobacter thiooxydans*	A0A1C2FZQ8	yes
A0A401JHY3	dabA SFMTTN_3545	*Sulfuriferula multivorans*	A0A401JI09	yes
A0A4R8IU21	dabA EDC23_1865	*Thiohalophilus thiocyanatoxydans*	A0A4R8IK62	yes
A0A543Q3U1	dabA DLNHIDIE_00855	*Acidithiobacillus thiooxidans* ATCC 19377	A0A543Q3T7	yes
A0A656HG06	dabA Thini_1858	*Thiothrix nivea* (strain ATCC 35100 / DSM 5205 / JP2)	A0A656HC39	yes
A0A6I6D7R3	dabA GM160_03865	*Guyparkeria halophila*	A0A6I6CXL2	yes
A0A7C9P570	dabA GZ085_05225	*Sulfuriferula multivorans*	A0A7C9K1R7	yes

A0A7T4WF07	dabA H2515_03250	*Acidithiobacillus ferrivorans*	A0A1B9BXJ4	yes
A0A845UBV2	dabA GL267_05340	*Acidithiobacillus ferrianus*	A0A845U389	yes
A0A8I1MUZ6	dabA J0I24_06505	*Thiomonas arsenitoxydans* (strain DSM 22701 / CIP 110005 / 3As)	A0A8I1STW5	yes
A0ABS5ZQJ3	dabA HJG40_08035	*Acidithiobacillus concretivorus*	A0ABS5ZRF7	yes
A0ABS5ZVG3	dabA HAP95_02765	*Acidithiobacillus sulfurivorans*	A0ABS5ZVA8	yes
A0ABU6FR22	dabA OW717_10680	*Acidithiobacillus ferriphilus*	A0ABU6FRH 9	yes
A0ABV0EK15	dabA V6E02_11075	*Thiobacter aerophilum*	A0ABV0EK25	yes
A0ABV4TZC3	dabA ACERLL_16645	*Thiohalorhabdus methylotrophus*	A0ABV4TYQ 5	yes
A0A512LCI1	dabA, TPL01_32400	*Thiobacillus plumbiphila*	A0A512LC84	yes
A0ABZ0YYS3	dabA SR882_04640	*Guyparkeria halophila*	A0ABZ0YYU6	yes
A0ABZ1CMB 8	dabA VA613_13940	*Thiobacillus sedimenti*	A0ABZ1CI67	yes
A4BLJ5	dabA NB231_15223	*Nitrococcus mobilis* Nb-231	A4BLJ3	yes
C4FIQ8	dabA SULYE_0448	*Sulfurihydrogenibium yellowstonense SS-5*	C4FIQ7	yes-fused
D0KZ77	dabA1 Hneap_0907	*Halothiobacillus neapolitanus* (strain ATCC 23641 / DSM 15147 / CIP 104769 / NCIMB 8539 / c2) *(Thiobacillus neapolitanus)*	D0KZ79	yes
D3DIA8	dabA HTH_1102	*Hydrogenobacter thermophilus (strain DSM 6534 / IAM 12695 / TK-6)*	D3DIA7	yes, fused
D3SP01	dabA Thal_0253	*Thermocrinis albus (strain DSM 14484 / JCM 11386 / HI 11/12)*	D3SP02	yes, fused
D5X328	dabA Tint_0113	*Thiomonas intermedia (strain K12) (Thiobacillus intermedius)*	D5X326	yes
D6CQJ7	dabA THI_0132 THICB1_110318	*Thiomonas arsenitoxydans (strain DSM 22701 / CIP 110005 / 3As)*	D6CQJ5	yes
G0JL81	dabA Acife_3126	*Acidithiobacillus ferrivorans SS3*	G0JL83	yes

H5SBX7	dabA HGMM_F07F09C38	*uncultured Aquificia bacterium*	H5SBX8	yes--fused
O67026	dabA aq_863	*Aquifex aeolicus (strain VF5)*	O67027	yes--fused
Q0AHW4	dabA Neut_0801	*Nitrosomonas eutropha (strain DSM 101675 / C91 / Nm57)*	Q0AHW6	yes
A0A1A6C0K6	dabA Thpro_022334	*Thiobacillus prosperus (strain DSM 5130/JVM 30709)*	A0A1A6C0J5	yes
Q1QFS7	dabA1 Nham_4323	*Nitrobacter hamburgensis (strain DSM 10229 / NCIMB 13809 / X14)*	Q1QFS5	yes
Q3SFK3	dabA Tbd_2653	*Thiobacillus denitrificans (strain ATCC 25259 / T1)*	Q3SFK1	yes
A0A238D7K2	dabA THIARS_70938	*Thiobacillus delicatus (strain DSM 5494)*	A0A238D7N1	yes
Q3SR42	dabA1 Nwi_1990	*Nitrobacter winogradskyi (strain ATCC 25391 / DSM 10237 / CIP 104748 / NCIMB 11846 / Nb-255)*	Q3SR40	yes
W0DCR8	dabA THERU_06545	*Thermocrinis ruber*	W0DH74	yes--fused
W9V6Y6	dabA D779_1733	*Imhoffiella purpurea*	W9VGL4	yes

Movie S1 (separate file). Remodeling of the lid region enclosing the active site (residues 474-509) and coordinated movement of DabB (residues 333-346).
